# From Sustainability Narratives to Digital Infrastructures: Mapping the Transformation of Smart Agri-Food Systems

**DOI:** 10.3390/foods15030469

**Published:** 2026-01-29

**Authors:** Alina Georgiana Manta

**Affiliations:** Faculty of Economics and Business Administration, University of Craiova, 200585 Craiova, Romania; alina.manta@edu.ucv.ro

**Keywords:** digital innovation, smart agri-food, sustainability transitions, circular economy, knowledge systems

## Abstract

The convergence of digital innovation and sustainability imperatives is transforming the architecture of agri-food systems, signaling not just a technological upgrade, but a reorganization of how food production, distribution, and governance are approached. This study presents a comprehensive bibliometric mapping of global research on sustainable and digital agri-food systems between 2004 and 2025, based on data from the Web of Science Core Collection and analyzed using the Bibliometrix within RStudio (Version: 2024.12.1+563). Through co-word analysis, bibliographic coupling, and temporal trend exploration, the study identified a marked surge in scholarly activity after 2020, driven by the alignment of digital innovation with major policy frameworks such as the European Green Deal and the Farm-to-Fork Strategy. Findings highlight Europe—particularly Italy, the Netherlands, and France—as the leading knowledge hub, demonstrating both institutional capacity and policy responsiveness. Thematic clusters revealed four dominant trajectories in recent research: digital governance, blockchain and traceability, circular economy integration, and ESG-based performance frameworks. These directions suggest a transition from narrow efficiency-centered approaches to more comprehensive, ethically informed, and technologically integrated agri-food systems. The study frames digitalization as both a technical infrastructure and a socio-organizational driver that reshapes transparency, accountability, and coordination within food value chains. It also outlines strategic entry points for improving interoperability, bridging digital divides, and advancing collaborative governance models across the agri-food sector. In addition to its empirical findings, the article contributes methodologically by positioning bibliometric analysis as a valuable tool for tracking major conceptual and structural shifts within food system research. In conclusion, digital transformation in agri-food systems is not merely about technological enhancement—it is a fundamental restructuring of processes, relationships, and governance mechanisms that define how food systems operate in an era of innovation, complexity, and sustainability challenges.

## 1. Introduction

The global agri-food system is undergoing a rapid digital transformation, often promoted as essential for meeting sustainability challenges. By 2050, the world’s population is projected to reach over 9 billion, requiring a 60–70% increase in food production even as current agriculture already contributes roughly one-quarter of anthropogenic greenhouse gas emissions [1]. Digital technologies—from precision farming and AI-driven decision tools to sensor networks and blockchain—are heralded as potential “game-changers” to produce more with less environmental impact [2]. This enthusiasm has given rise to the notion of a “digital innovation” in agri-food systems, frequently framed as part of a fourth agricultural revolution or “Agriculture 4.0” [3,4]. Proponents describe an ongoing paradigm shift towards data-driven, smart agriculture: a radically new model of food production where real-time data and automation optimize resource use and sustainability outcomes [5,6]. High-level policy narratives echo this optimism. International agencies and governments around the world portray digitalization as a key catalyst for sustainable intensification and improved food system resilience, aligning technological innovation with the pursuit of global food security and environmental goals [2,7].

However, while digital innovation is frequently framed as a catalyst for transformation, much of the current discourse remains confined to a symbolic rather than substantive digitalization, offering rhetorical commitment rather than systemic integration. A growing body of research cautions that the purported benefits of digital agriculture may be inflated by hype and optimistic speculation rather than grounded in consistent empirical evidence [4,8]. On the one hand, a dominant narrative in both industry and policy circles envisions digital tools as an unequivocal public good, capable of maximizing yields and efficiency in line with the Green Revolution. Lajoie-O’Malley et al. [7], for example, found that major international institutions—the World Bank, Food and Agriculture Organization of the United Nations (FAO), Organization for Economic Co-operation and Development (OECD)—imagine digitalization primarily to uphold and enhance the existing industrial agri-food system, prioritizing productivity gains based on the controversial assumption that increasing output automatically improves food security. On the other hand, critics argue that these visions may amount to a policy illusion. Rather than transforming the agri-food paradigm, digitalization could be reinforcing the status quo power structures and agribusiness-dominated model of agriculture [8,9]. Indeed, some observers note that what is billed as a “digital revolution” might instead represent a continuation—even a deepening—of the prevailing productivity regime, with new technologies entrenching the market dominance of a few large agri-food corporations. In this view, far from democratizing food systems, digital innovation risks reproducing existing inequities, unless checked by critical governance and inclusive innovation practices [3,10]. The question thus arises: is digitalization in agriculture genuinely driving a conceptual transformation in how we understand and pursue sustainability, or is it largely a rhetorical device in policy that masks business-as-usual?

Mounting empirical evidence gives credence to the skeptical perspective. While digital farming platforms, big data analytics, and AI-driven tools promise efficiency and sustainability gains, their on-the-ground impacts have so far been mixed and context-dependent [4]. For instance, a recent analysis emphasized that many technological applications remain in trial phases with inconclusive effects on ecosystem services and resource use [7]. The initial enthusiasm surrounding precision agriculture and farm management software has often outpaced demonstrable outcomes, leading some scholars to characterize digital agriculture as being at the “peak of inflated expectations” in the classic technology hype cycle [4]. Clapp and Ruder [8] noted that corporate claims about digital tools—for example, that precision techniques will drastically reduce chemical inputs—are not yet backed by robust evidence, and they warn of a disconnect between marketing narratives and reality. At the same time, academic and civil society critics have raised deeper questions about who truly benefits from digital innovation. There is growing concern that digitalization may be empowering incumbent agri-food giants who control data infrastructures, reinforcing oligopolistic market power rather than empowering farmers or consumers [8,11]. Prause [9] observes that digitalization, as currently unfolding, tends to “accelerate the industrialization of agriculture” by embedding farms into corporate-controlled data platforms and input supply chains, thereby extending corporate influence across the entire food supply web. In short, these critiques cast doubt on the narrative of an egalitarian tech-driven paradigm shift, suggesting instead that digital agriculture may perpetuate existing paradigms under the illusion of innovation.

Moreover, numerous socio-institutional challenges accompany the technical aspects of digital agriculture’s rise. Researchers are increasingly examining issues of data governance, farmer agency, and ethical implications of smart farming [12,13]. Data ownership, access, and privacy have emerged as pivotal concerns: farmers are often wary about who collects and controls their farm data, how it will be used, and whether they will share in the value it generates [14]. Early studies document a “trust gap”, with many smallholders reluctant to adopt data-intensive technologies due to fears of surveillance or unfair benefit distribution [15]. Power dynamics in the agri-food chain are also being scrutinized. Digital platforms can shift bargaining power upstream or downstream, potentially marginalizing smaller actors. Scholars have noted the risk of widening digital divide, wherein well-resourced farms and agribusinesses leverage advanced analytics while resource-poor farmers are left behind, exacerbating inequality [11,16]. Bronson [12] applies a responsible innovation lens to highlight how current designs of farm management software often overlook the needs of diverse farming communities, resulting in uneven engagement and benefits. Labor and social implications are another important facet. Automation and decision-support AI could indeed alleviate some farm labor and improve precision, but they may also deteriorate farm work, increase the surveillance of farm workers, or displace certain labor segments [9]. Prause [9] warns that without safeguards, digital farming may introduce new forms of labor control and exacerbate precarity for farm workers, undercutting the social pillar of sustainability. In sum, alongside the technical promise of digital tools, there is a parallel discourse interrogating the socio-economic transformations (or lack thereof) engendered by these tools—a discourse that asks whether we are witnessing a profound paradigm shift towards more equitable, knowledge-intensive food systems or simply a high-tech extension of existing industrial paradigms.

Against this backdrop, a clear *research gap* has emerged in the literature. While there is abundant research on individual digital innovations (e.g., studies on blockchain for traceability, AI for crop modeling, or IoT sensor networks on farms) and an increasing number of relevant essays on governance and ethics, few studies have attempted to synthesize these threads to determine whether, taken together, they amount to a transformative shift. The current scholarship remains fragmented: on the one hand, technocentric studies extol digital tools’ potential sustainability benefits [17,18], and on the other, critical social science works raise alarms about digitalization’s pitfalls [8,19]. What is missing is a holistic overview of the evolving knowledge ecosystem at the intersection of digital technology and sustainable agri-food systems. In other words, the literature lacks a comprehensive mapping of how digitalization is being studied in the context of sustainable agriculture and food—what the main focal points are, how they have evolved over time, and to what extent this body of work supports the notion of a paradigm shift versus a policy illusion. Addressing this gap is important not only for academic understanding, but also for guiding policy: if digitalization is predominantly a policy buzzword unsupported by transformative change on the ground, resources could be misallocated and truly novel solutions overlooked [10]. Conversely, if there is evidence of an emerging paradigm—for instance, a convergence of research and practice around agroecological digital tools or participatory data commons—identifying it early on is crucial for nurturing its development.

Furthermore, despite the substantial growth of academic production in recent years, the literature remains fragmented and unevenly distributed across regions and institutions. *A major research gap* also lies in the absence of longitudinal and comparative analyses that can explain the dynamics of knowledge production over time. Many previous studies have treated bibliometric data as static snapshots, neglecting the complex temporal relationships that characterize evolving research ecosystems. Additionally, limited attention has been paid to the interplay between institutional productivity, international co-authorship, and citation performance. Another underexplored dimension concerns the diffusion of emerging themes—such as big data, blockchain, and digital transformation—in connection with the broader discourse on sustainability. This study seeks to address these deficiencies by adopting a dynamic, integrative framework capable of mapping the structural and temporal evolution of scientific production.

The *purpose of this article*, therefore, is to examine whether the digital innovation in agri-food systems represents an authentic paradigm shift or a superficial policy narrative. We approach this question by conducting a bibliometric and content analysis of the sustainable agri-food literature spanning 2004–2025, a period covering the rise of digital agriculture discourse. By mapping over two decades of research outputs, we aim to identify major knowledge clusters and trends that characterize the nexus of digitalization and sustainable food systems. In particular, our study performed a bibliometric mapping to reveal how topics have coalesced or diverged over time, shedding light on the intellectual structure of this interdisciplinary field. The analysis uncovers several prominent research clusters that have arisen during this period. The first cluster consistently highlights blockchain’s promise for strengthening provenance tracking, reducing fraud, and even advancing sustainability goals by enabling more transparent and efficient logistics [20]. A second cluster involves the integration of Environmental, Social, and Governance (ESG) principles and metrics into agri-food enterprises and investment. This line of research reflects a broader trend of linking digital agriculture with corporate sustainability performance and finance. For example, recent work has examined how agribusiness firms’ ESG ratings correlate with their resource efficiency and market outcomes [21]. The global focus on ESG has intensified alongside the Sustainable Development Goals and climate commitments, making ESG a significant framework for assessing agri-food system transformation. A third cluster focuses on circular economy and resource circularity in food systems. Here, scholars investigated how digital innovations (such as precision farming, waste tracking apps, or online platforms for food sharing) can facilitate circular practices like waste reduction, recycling, and nutrient cycling in agriculture [1]. The circular economy is increasingly proposed as a new paradigm to replace linear “take-make-dispose” food supply chains with regenerative, closed-loop models. Our analysis traces how concepts of circular agri-food systems, often enabled by digital tools for monitoring and coordination, have gained traction in the literature over the study period.

Therefore, *the study’s contributions* are both theoretical and methodological. Theoretically, it substantiates the proposition that the digital transformation of agri-food systems constitutes a multi-level adaptive process—one shaped by the recursive interplay between technological infrastructure, institutional coordination, and socio-environmental imperatives. The observed co-evolution of digital and green transitions supports the notion of a “twin transition”, as articulated by Finotto et al. [22], but reframes it through a global bibliometric lens. Methodologically, this research advances the use of bibliometric coupling, co-word, and trend analyses as integrative tools for mapping scientific evolution across disciplines. By triangulating these methods, it becomes possible to detect not only thematic coalescence but also intellectual drift, revealing the latent dynamics of influence, collaboration, and paradigm consolidation that conventional reviews often overlook.

Therefore, this study addresses the following research questions:

*RQ1.* How have the central themes in the literature on sustainable agri-food systems evolved with the integration of digital technologies, and what do these thematic shifts reveal about the conceptual transformation of the field?

*RQ2.* To what extent do scholarly collaboration patterns reflect the emergence of thematic knowledge clusters—such as those focused on digital governance, traceability, circular economy, or ESG performance—and how do these clusters contribute to the interdisciplinary consolidation of digital agri-food research?

*RQ3.* How do institutional affiliations and research centers differ in their emphasis on digitalization and sustainability, and what role do these institutions play in shaping the global research agenda on agri-food systems?

*RQ4.* How does the geographical distribution of research output and international cooperation reflect regional policy frameworks and capacity for digital innovation in agri-food transformation?

*RQ5.* What directions should future scholarships pursue to develop an integrated analytical framework that connects digital infrastructures, sustainability transitions, and socio-institutional change in agri-food systems?

The structure of the paper follows a logical progression that reflects both the analytical scope and thematic depth of the study. Section 1 introduces research by situating the digital transformation of agri-food systems within the broader context of sustainability imperatives and paradigm shift debates. Section 2 engages with the existing literature, mapping the evolution of scholarly discourse on agri-food sustainability, the increasing integration of digital technologies, and the intersections between these two domains. Section 3 outlines the methodological design, detailing the bibliometric tools and analytical techniques applied to trace the field’s development. Section 4 presents the core empirical findings, including the identification of dominant thematic clusters and the intellectual, institutional, and geographic contours of the research landscape. Section 5 interprets these results through a critical lens, addressing the central question of whether the observed patterns represent a substantive transformation or reflect a more symbolic policy narrative. Finally, Section 6 draws together the key insights of the study and proposes directions for future research aimed at fostering inclusive, ethically guided, and digitally enabled sustainability transitions in agri-food systems.

## 2. Literature Review

The accelerating convergence between digital technologies and sustainable agri-food systems has triggered an integrated transformation across knowledge systems and policy instruments of how agricultural production, distribution, and consumption are conceived and managed. Over the last two decades, the “digital innovation” in the agri-food domain has been hailed as a pathway toward efficiency, transparency, and ecological balance. Yet, as this study suggests, that narrative often masks deep contradictions between technological optimism and uneven sustainability performance. Drawing on bibliometric data from Web of Science (2004–2025) and critical interpretive analysis, this paper interrogates how digital innovation—manifested through artificial intelligence (AI), the Internet of Things (IoT), blockchain, digital twins, data analytics, and tokenization—has reshaped the organizational, institutional, and ecological architectures of agri-food systems. Using Bibliometrix for structural mapping, the analysis exposes not only exponential publication growth after 2015 but also fragmentation, regional asymmetries, and a still embryonic integration of digital affordances with sustainability governance.

The early phase of the literature (2004–2015) reflected a cautious techno-economic orientation dominated by precision agriculture, automation, and early ICT adoption, rarely engaging with sustainability as a systemic construct. However, from 2016 onwards, the intersection between digital transformation and sustainable food systems became a central scholarly concern, accelerated by global disruptions, most notably climate change, supply chain volatility, and the COVID-19 pandemic [23]. Digital agriculture was reframed as a governance instrument to reconcile productivity with resource efficiency, circularity, and social inclusion. However, despite the discursive integration of sustainability, empirical validation remained partial, often confined to case studies or conceptual frameworks.

### 2.1. The Digital-Sustainability Nexus: From Efficiency Narratives to Systemic Integration

Contemporary literature identifies digitalization as both an enabler and a disruptor of sustainable agri-food transitions. Scholars argue that digital infrastructure like sensor networks, data analytics, and AI-driven decision systems enhance traceability, reduce food loss and waste (FLW), and optimize resource allocation [24]. Their systematic review of Industry 4.0 technologies revealed that despite the broad promise of digital solutions, empirical evidence of their effectiveness in preventing FLW remains fragmentary. The authors constructed a research agenda emphasizing contextual differences, governance, and sustainability as underexplored dimensions. Similarly, Sica et al. [25] contend that digital technologies are instrumental to Life Cycle Assessment (LCA) empowerment, enabling real-time data flows that can support circular economy transitions. However, their scenario analysis uncovered major barriers—data heterogeneity, regulatory gaps, and limited managerial readiness—that temper the transformative potential of “empowered LCAs”.

In parallel, the discourse on digital transformation in agri-food cooperatives underscores the social and institutional dimensions of innovation. Guzmán et al. [26] outline that digital maturity in cooperatives correlates strongly with knowledge-sharing intensity, leadership support, and access to funding, suggesting that sustainability outcomes are not technologically determined but institutionally mediated. Their work, situated in the Andalusian cooperative ecosystem, exposes the uneven diffusion of digital technologies in peripheral regions, where social capital and cooperative governance substitute for absent infrastructure. This aligns with Ciasullo et al. [27], who reconceptualized digital transformation as a capability-based process. Using a survey of 300 Italian SMEs, they showed that big data analytics capabilities (BDAC) and digital capabilities (DC) jointly foster a data-driven culture that, in turn, accelerates digital transformation. Their covariance-based SEM approach evidence that technological adoption without cultural transformation fails to deliver systemic change—an important insight for sustainability policy.

### 2.2. Traceability, Transparency, and Blockchain Narratives

A dominant stream in the literature positions blockchain and distributed ledger technologies as the backbone of traceability, trust, and circularity in global agri-food supply chains. Charlebois et al. [28] provide a comparative assessment of digital traceability across OECD countries, revealing heterogeneous regulatory architectures and variable consumer trust dynamics. Their study highlights how national policy frameworks, especially those related to food safety and transparency, shape the scalability of digital traceability systems. Zarbà et al. [29] advanced this perspective through a systematic literature analysis employing PRISMA and VOSviewer (version 1.6.19), concluding that blockchain’s innovative role in agri-food systems lies more in institutional credibility and anti-fraud assurance than in purely technical optimization. However, they caution that many blockchain pilots remain descriptive, lacking a quantitative evaluation of sustainability outcomes.

A complementary conceptual framework emerged from van Wassenaer et al. [30], who introduced “tokenized circularity” as a mechanism to align information and value flows. Their analysis situates tokenization within the broader circular economy paradigm, demonstrating how blockchain-based tokens can incentivize collaboration and accountability in circular agri-food ecosystems. However, they warn that without standardized protocols and interoperable data layers, tokenization risks replicating silos rather than dismantling them. This insight reframes blockchain from a technical artifact into a socio-economic infrastructure whose governance determines its sustainability potential.

### 2.3. Circularity, Waste Valorization, and Digital Twins

The literature on circularity and agri-food waste management converges on a striking observation: while digital tools are widely invoked as sustainability enablers, empirical evidence of systemic waste reduction remains scarce. Campana et al. [31] performed a large-scale bibliometric and thematic review (373 publications, 2015–2025) and identified six major clusters—from predictive waste analytics to circular bioeconomy applications such as anaerobic digestion and pyrolysis. They emphasize that research productivity correlates with regional wealth, exposing a “sustainability divide” between high- and low-income countries. Their findings reinforce the argument that digital innovation functions as both an operational instrument and a structural amplifier of inequalities when unaccompanied by inclusive governance frameworks.

Complementary to waste analytics, the digital twin literature marks a methodological frontier for integrating physical and digital processes. Melesse et al. [32,33,34] jointly established the conceptual foundations of agri-food digital twins (AFDTs). Their systematic reviews and interoperability studies suggest that digital twins can simulate perishable supply chains in real-time, improving resource allocation, planning, and risk management. However, they also document the nascency of this research stream: most implementations remain at the conceptual or pilot stage, disconnected from legacy systems and sustainability KPIs. The promise of visibility thus confronts the reality of fragmented architectures and data asymmetries. As they persuasively note, visibility without governance is surveillance, not sustainability.

### 2.4. Innovation Ecosystems, Startups, and Digital Entrepreneurship

The entrepreneurial ecosystem literature advances another dimension of digital innovation in agri-food systems—its capacity to reconfigure market structures. Ranjbari et al. [35] provided a typology of 79 circular economy startups, distinguishing between biotech-driven sustainable producers, digital circular economy (CE) enablers, and circular packaging pioneers. Their two-tiered content and cluster analysis illustrates how digital CE enablers act as platforms connecting stakeholders across the value chain, operationalizing reduction strategies through data-based matchmaking. In contrast, biotech startups innovate upstream with repurposing and upcycling, while circular packaging ventures dominate the downstream. This classification elucidates how digital innovation modifies not only production logics but also the power geometry of sustainability transitions. However, as Sippel and Dolinga [36] incisively argue, the influx of venture capital into “agri-food tech” has also financialized sustainability itself. Their qualitative analysis of investment imaginaries shows that digital agri-food technologies are narrated simultaneously as profitable opportunities and moral imperatives—a discursive fusion that legitimizes speculative accumulation while obscuring environmental trade-offs. The authors thus unveil the political economy underpinning digital sustainability rhetoric: it produces value for investors as much as for ecosystems.

### 2.5. Digital Branding, Analytics, and Performance of Sustainability

An emergent stream situates digital innovation within marketing and consumer analytics, where big data metrics mediate value perception and sustainability signaling. Kanellos et al. [37] empirically linked desktop and mobile customer analytics to digital branding outcomes in agri-food firms, revealing strong correlations between analytics intensity and brand performance. In a parallel study, Kanellos et al. [38] explored profitability drivers across value chains, showing that efficient digital marketing can reconcile profitability with resource efficiency when guided by decision-support systems (DSSs). However, both studies implicitly expose a paradox: the same analytics infrastructures that enhance transparency and efficiency also deepen surveillance capitalism, where consumer data become the raw material of value creation. The digital sustainability agenda thus operates on a knife-edge—balancing visibility with privacy, optimization with autonomy.

### 2.6. Regional Asymmetries and the Uneven Geography of Digital Agriculture

Despite the global rhetoric of inclusiveness, the geography of digital innovation in agri-food systems remains profoundly uneven. Porciello et al. [23] provide one of the most comprehensive assessments for Africa, illustrating that although mobile penetration and platform deployment have expanded rapidly, evidence of transformative impact remains limited. They attribute this to structural barriers—costs, literacy, infrastructural deficits, and fragmented ecosystems—that constrain smallholders from leveraging digital agriculture fully. Their study aligns with Gusmanov et al. [39], who, focusing on rural Russia, developed a scenario forecasting model for agri-food development under digital economy conditions. Both highlight that digitalization amplifies preexisting inequalities unless supported by coordinated policy, institutional capacity, and investment in human capital.

Roeven et al. [40] pushed this argument further, interrogating digital agriculture as a socio-technical construct embedded in knowledge-based hierarchies. Their ethnographic collaboration between computer scientists and agri-food scholars exposes how abstraction—celebrated in computer science as a design virtue—can erase local knowledge and ecological specificity. By contrasting technical abstraction with the “disembedding” criticized in agri-food studies, they uncover the ideological work of openness and interoperability. Far from being neutral, digital infrastructures encode values and power relations that determine whose sustainability counts. This aligns with a growing reflexive turn in the literature that calls for interdisciplinary, governance-aware digitalization.

What the 2021–2025 corpus makes clear is that “digital innovation” in agri-food is not a rising tide that lifts all boats but a fragmented assemblage of infrastructures, governance logics, and managerial rhetorics whose sustainability payoff depends on who designs, owns, and steers them. At one pole sit solutionist narratives—digital twins, tokenized circularity, platformed services, photonics, AI/ML, 5G—promising traceability, precision, and decarbonization at scale [30,41,42,43,44]. At the other pole stand ethnographic and critical accounts warning that those same architectures can intensify enclosure, entrench vendor lock-in, and displace labor, even when draped in the language of openness and green transition [40,45]. Our bibliometric lens positions this review squarely between the two: neither anti-tech nor credulous, but insistent that “sustainable agri-food systems through digital innovation” is an empirical claim that must be evidenced—not declared.

To close the argument, two pivots are non-negotiable if “digital innovation” is to deliver sustainability rather than simply repackage efficiency rhetoric. The first is institutional: value creation must be demonstrably reallocated toward the weakest nodes (smallholders, workers, SMEs) through governance that binds technology to fairer market rules. Evidence across contexts shows that the same tools produce opposite outcomes depending on who controls interoperability, data rights, and standard setting. Blockchain’s promise becomes real only when embedded in credible audit regimes and aligned incentives along the chain [29,46,47], and tokenized circularity will remain a thought experiment without enforceable verification and partner inclusion [30]. The second pivot is capability formation: digital transitions stick when firms build the micro-foundations—managerial cognition, routines, and structures—that let them reconfigure assets, not when they merely procure apps or sensors [48,49]. Case-comparative work maps viable paths—purpose-driven, technology-exploration, and compliance-driven—yet also shows why many SMEs stall: weak absorptive capacity, ambiguous value propositions, and thin ecosystems [50,51].

## 3. Materials and Methods

Bibliometric analysis represents a scientifically validated approach for quantitatively and visually examining the structure and development of academic literature. Grounded in statistical logic and computational techniques, it enables researchers to uncover the intellectual architecture, thematic trajectories, and influential contributions within a given domain of inquiry [52,53,54,55,56,57,58]. Within fields such as agriculture and sustainability, this methodology facilitates the identification of foundational theoretical inputs, collaborative institutional networks, and emerging research frontiers that shape the evolution of the discipline [59,60]. The Bibliometrix package, developed in the R programming environment, serves as a powerful and internationally recognized platform for implementing advanced bibliometric investigations on datasets sourced from leading academic repositories, including Web of Science and Scopus [61].

Utilizing the RStudio environment as Bibliometrix within RStudio (Version: 2024.12.1+563) significantly enhances the methodological robustness of bibliometric investigations by ensuring both transparency and reproducibility across the analytical process [62]. It enables the automation of metadata processing, the construction of bibliometric performance indicators, and the generation of dynamic visualizations that reflect conceptual relationships within the field. Key functions within the Bibliometrix package—such as biblioAnalysis(), summary(), networkPlot(), and thematicMap()—facilitate in-depth exploration of co-authorship patterns, co-citation structures, and keyword co-occurrence networks, offering a systematic framework for identifying intellectual clusters and interdisciplinary linkages [63]. Complementing these capabilities, the Biblioshiny interface extends access through an intuitive graphical environment, supporting interactive exploration of publication output, citation impact, and thematic development with both analytical precision and visual clarity [64].

**Figure 1:**
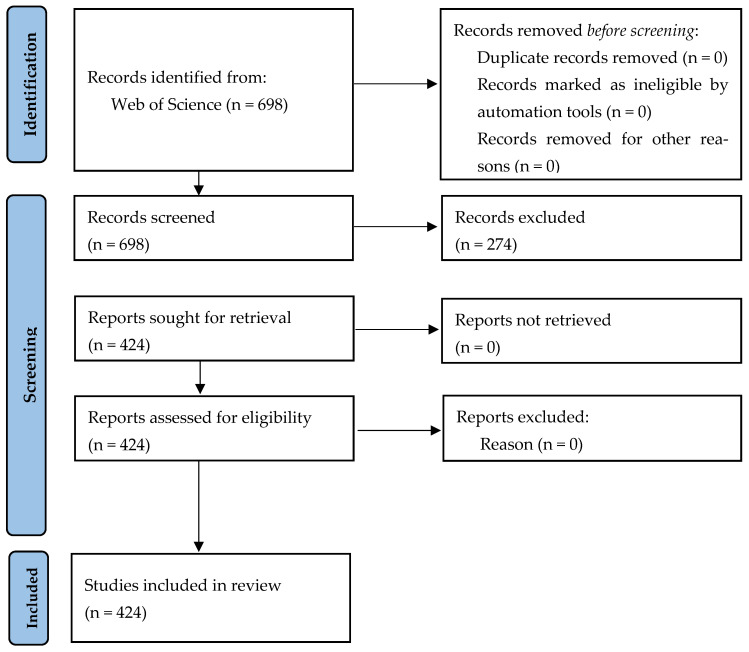
PRISMA flow diagram of the bibliometric search and screening process in Web of Science. Source: Own processing.

Together, these tools form a comprehensive and flexible analytical framework for the systematic mapping of scholarly production. By integrating Bibliometrix within RStudio, researchers are equipped to investigate complex scientific landscapes, trace global research trends, and discern dominant paradigms or knowledge gaps within any given field [65]. In the present study, the bibliometric process was structured in sequential stages to ensure methodological rigor (Figure 1). The initial phase involved querying the Web of Science Core Collection using targeted keyword combinations. These included “agri-food systems” in conjunction with “sustainable”, further refined by terms such as “digitalization” and “innovation” to sharpen the thematic scope. This process yielded a dataset comprising 698 documents related to agri-food system contexts, reflecting both the scale and thematic diversity of scholarly engagement with these issues.

**Figure 2:**
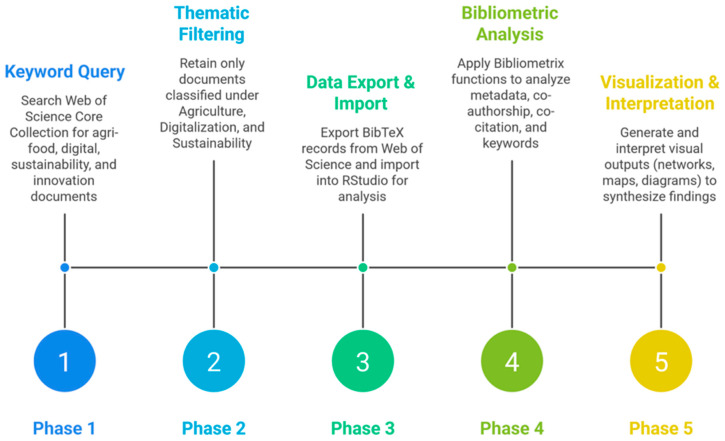
Bibliometric analysis workflow in Web of Science. Source: Own processing.

In the second phase of the analysis (Figure 2), thematic refinement was carried out to ensure the inclusion of literature directly aligned with the objectives of this study. From the initial corpus of documents retrieved, only those classified within the disciplinary categories of Agriculture Multidisciplinary, Food Science Technology, and Green Sustainable Science Technology were retained. This targeted filtering narrowed the dataset to 424 publications. Such delimitation allowed for a sharper analytical focus on scholarly contributions pertinent to the intersection of agri-food systems and digitalization. Notably, no temporal constraints were imposed during the selection process. This decision enabled the capture of the full chronological trajectory of research developments, thereby offering a longitudinal perspective on how the scholarly understanding of agri-food systems has evolved in response to digitalization stressors.

The third phase marked the transition from corpus curation to data processing. Bibliographic records were exported from the Web of Science Core Collection in BibTeX format and subsequently imported into the RStudio environment for analysis. This stage established the groundwork for quantitative bibliometric procedures. The application of the Bibliometrix package facilitated the systematic organization and interrogation of the metadata. A range of analytical functions was deployed to examine scholarly performance metrics, co-citation relationships, co-authorship patterns, and the co-occurrence of conceptual keywords. A total of 424 records underwent structured bibliometric evaluation.

In the final stage, attention shifted toward the interpretative synthesis of the results through visual and conceptual mapping. Drawing on the combined capabilities of the Bibliometrix suite and its Biblioshiny interface, the analysis produced a series of visual outputs, including keyword co-occurrence networks, author and institutional collaboration maps, and temporal diagrams of thematic evolution. These visualizations provided significant insight into the main currents of scholarly inquiry on agri-food systems and illuminated how intellectual trajectories have been shaped by digitalization disruptions over time.

## 4. Results

### 4.1. Descriptive Bibliometric Results

**Figure 3:**
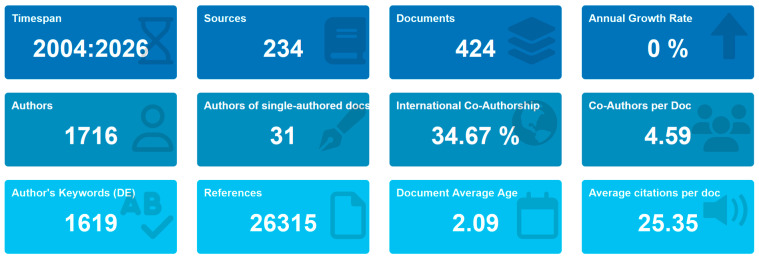
Overview of the bibliometric structure of the analyzed literature. Source: Own processing in Bibliometrix.

The quantitative synthesis presented in Figure 3 encapsulates the structural dynamics and productivity indicators of the analyzed corpus between 2004 and 2025 (some studies appear with a 2026 year of publication due to difference between the online publication year and the printed (issue/volume) publication year), offering an empirical overview of the field’s evolution. Over this twenty-one-year period, the dataset comprises *424 documents* disseminated across *234 distinct sources*, highlighting both the multidisciplinary and the dispersal of scholarly output. This distribution suggests that research on the digital–sustainability interface in agri-food systems has yet to reach stabilization, as knowledge is still being co-produced across heterogeneous disciplinary domains, including agricultural sciences, information systems, and sustainability studies.

The *annual growth rate of 0%* may appear counterintuitive given the exponential publication surge observed between 2018 and 2024 in the preceding figures. However, this apparent stagnation can be interpreted not as a contraction but rather as a sign of epistemic maturity, where the thematic field transitions from exploratory expansion to conceptual consolidation. In emerging interdisciplinary research, such plateauing often denotes the establishment of shared theoretical frameworks and methodological alignment, rather than diminishing relevance.

The authorship structure further substantiates this pattern of maturation. With *1716 contributing authors* and an average of *4.59 co-authors per article*, the field emphasizes a pronounced culture of collaboration. This indicator exceeds the average co-authorship rate typically reported in sustainability research (3.5–3.8), reflecting the inherently cross-sectoral nature of digital sustainability research that integrates academic, industrial, and policy perspectives. Notably, international co-authorship accounted for 34.67% of all publications, confirming that over one-third of contributions are products of transnational research partnerships. This metric places the field above the global average of 27% reported for environmental and technological studies, suggesting an increasingly interconnected and globally distributed knowledge ecosystem.

In contrast, only *31 single-authored publications* were identified, representing a marginal proportion of the total corpus. This distribution underscores that knowledge creation is driven by inter-institutional consortia and project-based networks rather than individual scholarship. The bibliometric structure thereby mirrors the governance complexity of the agri-food sector itself, which relies on multi-actor collaboration and distributed expertise.

The conceptual heterogeneity of the field is reflected in the presence of *1619 author-supplied keywords*, indicating a high level of thematic fluidity and innovation. Such lexical diversity reveals a dynamic research environment in which emerging constructs—such as *blockchain traceability*, *digital twins*, and *sustainable value chains*—continuously redefine the intellectual contours of the discipline.

Citation-based indicators reinforce the field’s growing academic influence. The *average citation rate of 25.35 citations per document* suggests above-average impact when compared with broader sustainability research benchmarks, which typically oscillate between 15 and 18 citations per article. This enhanced visibility is closely associated with the recency of publication, as reflected by a *mean document age of 2.09 years*, which indicates that the field’s most influential works have emerged within the last five years. The cumulative *26,315 references* cited across all documents—equivalent to an average of approximately 62 citations per article—signify a deeply anchored and theoretically informed research base, consistent with an area undergoing rapid conceptual refinement.

Therefore, these bibliometric descriptors portray a field that is thematically expansive, methodologically hybrid, and highly collaborative. The interplay between strong international connectivity and high citation density suggests that digital sustainability research in agri-food systems has transitioned into a phase of intellectual consolidation and theoretical diversification. While earlier phases (2004–2015) were characterized by dispersed exploratory inquiries, the current period (post-2020) appears to foster a more coherent research agenda centered on governance, ethical accountability, and the systemic integration of digital technologies.

### 4.2. Evolution of Scientific Output and Citation Impact over Time

**Figure 4:**
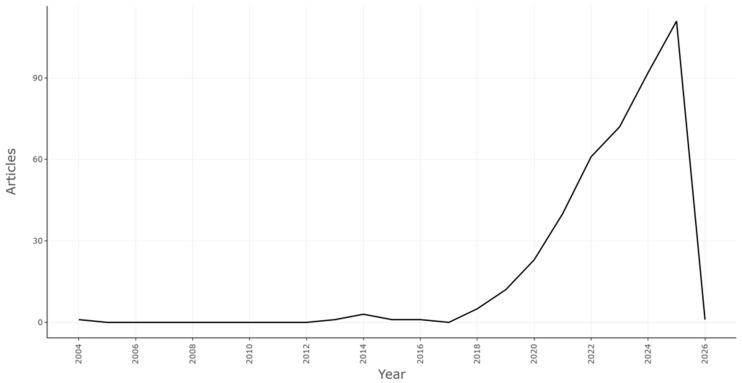
Evolution of Scientific Output. Source: Own processing in Bibliometrix.

**Figure 5:**
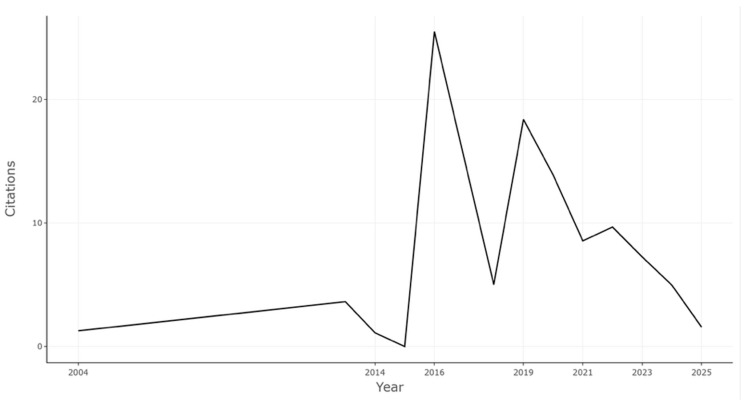
Citation Impact Over Time. Source: Own processing in Bibliometrix.

**Figure 6:**
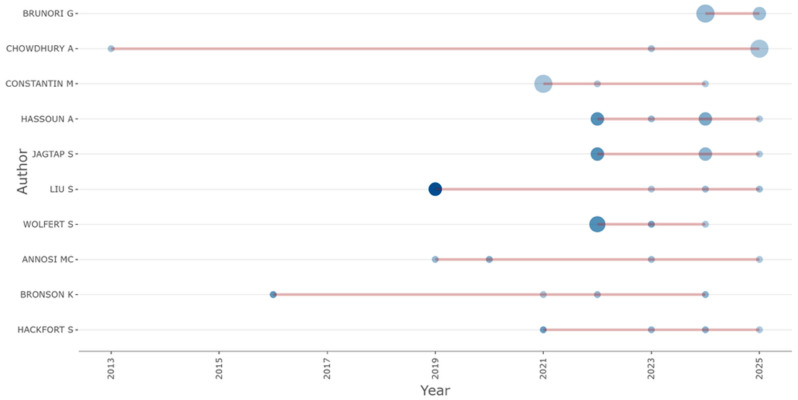
Authors’ production over time. Source: Own processing in Bibliometrix.

The temporal analysis of scientific production provides relevant insight into the developmental trajectory of research on digital transformation and sustainability within the agri-food sector. As illustrated in Figure 4, Figure 5 and Figure 6, the field exhibits a distinct three-phase evolution: an incipient phase of sporadic publication activity (2004–2014), a period of accelerated growth and thematic diversification (2015–2022), and a phase of consolidation and reflexive maturity (2023–2026).

During the *early stage (2004*–*2014)*, publication volumes remained negligible, with fewer than five annual outputs. This embryonic period reflects the nascent articulation of digitalization discourses within sustainability frameworks, when attention was primarily directed toward technological efficiency rather than systemic transformation. The average citation rates during this stage were modest, signaling limited cross-disciplinary engagement and the absence of an established research community.

The *second phase (2015*–*2022)* marked an inflection point characterized by exponential growth in both publication numbers and thematic diversity. The bibliometric trends coincide temporally with major international initiatives promoting digital agriculture and sustainability, such as the UN 2030 Agenda for Sustainable Development, the European Digital Strategy, and the FAO’s e-Agriculture framework. This alignment suggests that policy and funding mechanisms played a catalytic role in stimulating academic output. Indeed, annual scientific production rose sharply after 2019, corresponding with the diffusion of emergent technologies such as Internet of Things (IoT) applications, blockchain-based traceability, and big data analytics in agriculture.

Interestingly, the average citations per year exhibited a pattern of volatility rather than linear growth. Peaks in citation intensity around 2016 and 2019 corresponded to highly influential publications that redefined conceptual boundaries between digital innovation and sustainability governance. Nevertheless, the subsequent gradual decline in average citation frequency does not necessarily reflect a reduced impact but rather a temporal citation lag, as recently published works have yet to accumulate full recognition. The persistence of above-average citation rates across recent years underscores the field’s continued relevance and high citation half-life.

The figure showing the *authors’ production over time* (Figure 6) reinforces this dynamic, revealing the emergence of a stable cohort of prolific contributors—among them Brunori, Chowdhury, Constantin, Hassoun, and Wolfert—whose consistent publication trajectories have shaped the intellectual structure of the domain. The temporal overlap between these authors’ active periods suggests an increasingly networked community, where continuity of contribution and inter-authorship reinforce cumulative knowledge production. Notably, the proportional rise in co-authored papers since 2020 reflects the intensification of multi-institutional collaboration, an indicator of the field’s institutional consolidation and thematic specialization.

The *third and most recent phase (2023–2026)* appears to signal a plateau in publication growth, yet this stabilization should not be misinterpreted as a decline. Rather, it indicates a transition from expansion to consolidation, as the field attains theoretical maturity and begins to engage critically with its own conceptual foundations. The trend mirrors patterns observed in other maturing interdisciplinary domains, such as circular economy studies or environmental informatics, where initial exponential growth is typically followed by a phase of intellectual integration and critical reflexivity [66].

Overall, the temporal evolution of scientific production and citations reveals a trajectory of *quantitative acceleration followed by qualitative deepening*. The field has progressed from fragmented experimentation toward a coherent body of research centered on governance, ethical accountability, and data-driven sustainability. The observed citation dynamics further suggest that the domain’s influence extends beyond agriculture to broader discussions on digital ethics, environmental resilience, and socio-technical transitions. Consequently, the bibliometric evidence positions this research stream as a strategic interdisciplinary frontier, bridging technological innovation with sustainability governance in an increasingly data-intensive world.

### 4.3. Geographical and Institutional Distribution of Research

The spatial and institutional mapping of scientific output reveals the geopolitical and organizational architecture underpinning knowledge production in digital innovation for sustainable agri-food systems. The bibliometric evidence outlines a pronounced European leadership, supported by a growing presence from Asia and North America, thereby confirming the global diffusion and multi-polar nature of the field.

**Figure 7:**
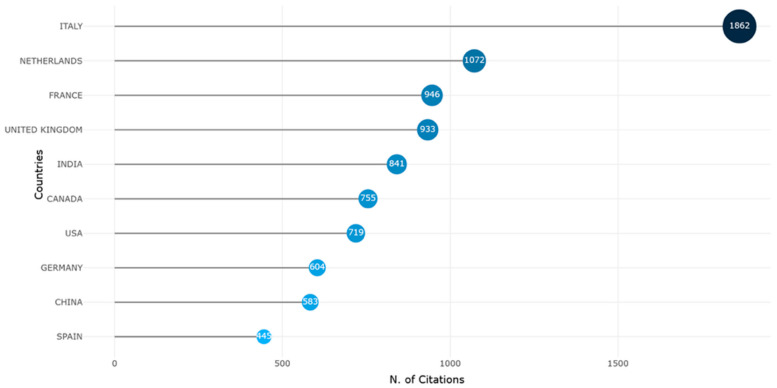
Country-level analysis. Source: Own processing in Bibliometrix.

The *country-level analysis* (Figure 7) indicates the geographic distribution of scholarly influence. The vertical axis lists countries, while the horizontal axis represents the number of citations attributed to publications affiliated with institutions in those nations. The size and shading of the blue markers enhance the visibility of relative citation strength.

The data revealed that Italy leads decisively, with 1862 citations, reflecting not only a high volume of publications but also their substantial academic impact. This aligns with Italy’s central role in shaping EU-level agricultural digitalization policy and research, particularly through frameworks such as the European Green Deal and its strong participation in Horizon Europe initiatives.

Following Italy, the Netherlands and France registered impressive citation counts of 1072 and 946, respectively. These countries are known for their integrated agricultural innovation systems and robust research infrastructures, often acting as incubators for interdisciplinary studies linking sustainability, food security, and technological adoption.

The United Kingdom, India, and Canada form a second tier of influential contributors, each exceeding 700 citations. Their prominence underscores the global nature of digital agriculture research, with India representing a key node in the Global South due to its scale, policy interest in smart farming, and increasing scientific visibility.

The United States, despite its established leadership in agri-tech and data-intensive agriculture, appears mid-ranking with 719 citations, possibly reflecting a more diversified or sectoral research output that disperses thematic focus across fields. Germany, China, and Spain round out the top ten, suggesting growing engagement but with relatively less concentrated impact.

Overall, the chart underscores a European research dominance in both productivity and influence while also highlighting emerging citation clusters in Asia and North America. It further suggests that citation metrics—beyond sheer publication volume—are important for evaluating knowledge leadership and agenda-setting capacity in the global digital agri-food landscape.

**Figure 8:**
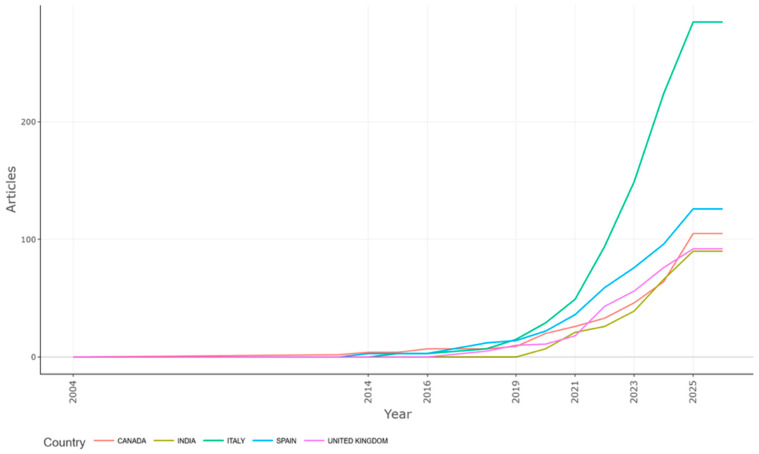
Temporal evolution of scientific production. Source: Own processing in Bibliometrix.

Figure 8 depicts the *temporal evolution of scientific production* in the field of digital agri-food systems across six leading countries—Canada, India, Italy, Spain, and the United Kingdom—over the period 2004 to 2025. The vertical axis represents the number of published articles, while the horizontal axis tracks the publication year. The data highlight a clear acceleration in research output starting around 2018, with a pronounced surge after 2020. This inflection point coincides with increasing global interest in digitalization as a response to sustainability challenges and systemic disruptions, including those triggered by the COVID-19 pandemic.

Among the countries analyzed, Italy stood out as the undisputed leader, exhibiting a steep and continuous growth trajectory that surpassed 250 articles by 2025. This reflects both policy-driven momentum—such as the European Green Deal and Farm-to-Fork Strategy—and robust institutional involvement in digital transformation research. Spain and the United Kingdom showed parallel upward trends, although at a more moderate scale, indicating their stable but comparatively restrained engagement. These trajectories suggest active participation in European digital agriculture discourse, though with varying institutional intensity.

India’s curve revealed a later but notable rise, particularly after 2021, reflecting the country’s growing prioritization of smart farming solutions, rural digitization programs, and food security initiatives within its national development strategy. Canada displayed a similar growth pattern to India, suggesting strong academic engagement and innovation-driven participation, albeit with a more gradual incline.

Overall, the figure underscores a global consolidation of research interest, with a strong European lead and increasing visibility of countries from both the Global North and Global South. The post-2020 acceleration across all countries suggests that digital agri-food systems have shifted from a niche research area to a central pillar in the sustainability and innovation agenda, with national research ecosystems aligning in response to both policy incentives and technological imperatives.

**Figure 9:**
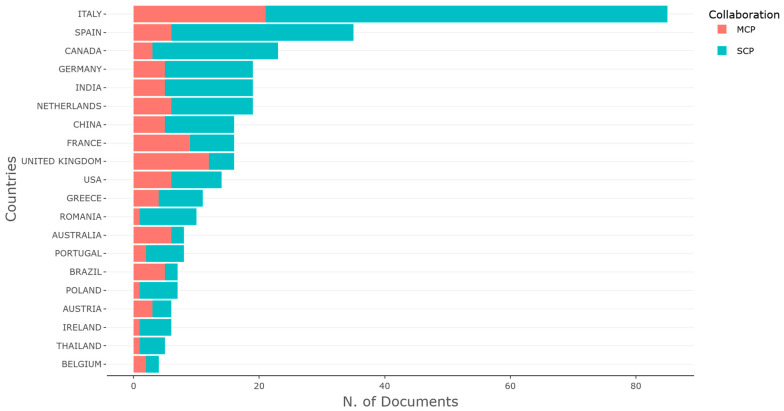
Corresponding authors’ countries and their distribution in single-country publications (SCPs) and multi-country collaborations (MCPs). Source: Own processing in Bibliometrix.

Collaboration dynamics, as illustrated in the *corresponding authors’ countries* figure (Figure 9), reinforce this spatial interpretation. This figure illustrates the distribution of scientific output by country in the field of digital transformation in agri-food systems, distinguishing between single-country publications (SCPs) and multi-country collaborations (MCPs). Italy leads the global research landscape both in terms of volume and the predominance of nationally led studies, reflecting a strong domestic research capacity supported by policy-driven initiatives such as the Farm-to-Fork Strategy and the European Green Deal.

Spain and Canada also exhibited high productivity, though Canada showed a greater proportion of internationally co-authored work, highlighting its integrative approach to transnational scientific collaboration. Germany, India, and the Netherlands followed closely, each displaying balanced engagement in both SCP and MCP, suggesting robust national infrastructures as well as openness to cross-border academic networks.

Countries such as France, the UK, and the USA outlined moderate output, with a relatively higher share of multi-country papers, indicating that their influence is often exerted through global partnerships rather than domestic research concentration. Meanwhile, emerging contributors like Romania, Poland, and Thailand, though lower in total volume, showed a strategic reliance on international collaboration as a vehicle for embedding themselves in the global knowledge system.

Overall, the chart revealed not only the geographic hubs of digital agri-food research but also the collaborative dynamics that shape the field’s conceptual structure. The prevalence of SCPs in leading European countries reflects internal research coherence, while the MCP patterns signal a widening of international scientific networks essential for addressing the complex and transboundary challenges of agri-food digitalization.

**Figure 10:**
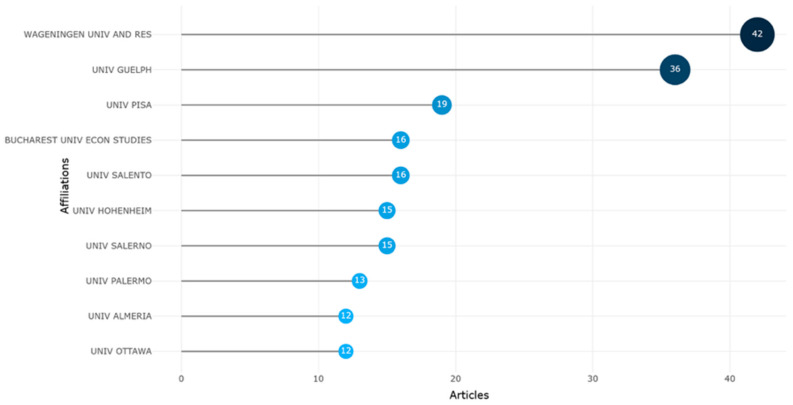
Most relevant affiliations. Source: Own processing in Bibliometrix.

**Figure 11:**
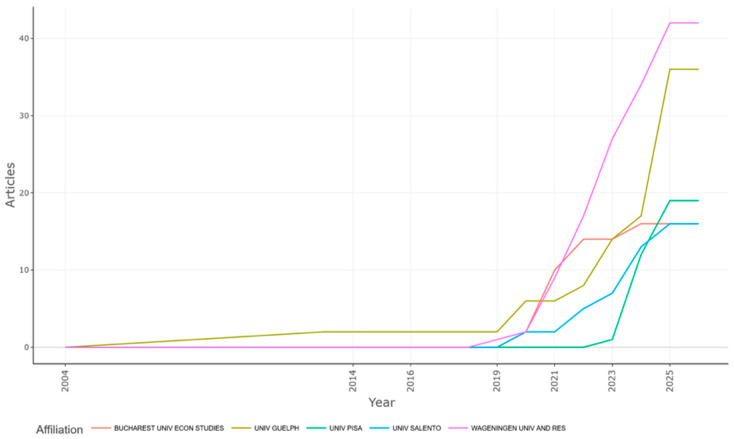
Affiliation production over time. Source: Own processing in Bibliometrix.

Institutional productivity data provide further granularity to this geographical structure. The *most relevant affiliations* (Figure 10) and *affiliation production over time* (Figure 11) plots identified Wageningen University & Research (Netherlands) and the University of Guelph (Canada) as the principal institutional anchors of global research. Their enduring prominence can be attributed to their long-standing expertise in agri-food sustainability and digital innovation. The University of Pisa and Bucharest University of Economic Studies also indicated substantial recent growth, reflecting the emergence of Southern and Eastern European universities as significant nodes in the scientific network.

The dynamic expansion of Wageningen University, in particular, aligns with its strategic integration of sustainability science and agricultural informatics, positioning it as a “boundary institution” where digitalization and ecological systems thinking intersect [13]. Meanwhile, the University of Salento’s rapid ascent since 2022 illustrates the diffusion of research capacity across previously peripheral regions, indicating a progressive democratization of knowledge production within Europe.

While collaboration remains concentrated in a few high-income regions, the increasing representation of institutions from Romania, Greece, and Portugal reflects a trend toward regional diversification. These emerging actors contribute context-specific insights, particularly in relation to smallholder digital adoption and sustainability transitions in semi-peripheral economies. Nevertheless, the absence of robust representation from Africa and South America remains a significant research gap, constraining the field’s global inclusiveness and limiting its capacity to address geographically diverse agricultural realities.

In synthesis, the geographical and institutional bibliometric evidence depicts a polycentric yet unequal scientific landscape, characterized by strong European dominance, expanding Asian engagement, and persistent southern underrepresentation. The evolving interplay between traditional research powerhouses and emerging regional actors illustrates a broader shift from concentration to distributed collaboration, a transformation that mirrors the decentralizing logic of digital innovation itself. In this sense, the field’s structure not only reflects global inequalities but also embodies its own aspiration toward transnational, systemic sustainability.

### 4.4. Intellectual and Conceptual Structure of the Field

The intellectual and conceptual structure of research on sustainable agri-food systems through digital innovation revealed a complex and dynamic interplay between technological advancement, sustainability imperatives, and systemic transformation. The co-word and factorial analyses indicate that the field’s conceptual core has progressively evolved from technology-centric inquiries toward integrative models that combine social, economic, and ecological perspectives. This transformation marks the transition from isolated digitalization frameworks to the emergence of an interdisciplinary paradigm that positions digital technologies as enablers of sustainability-oriented innovation rather than mere instruments of efficiency.

**Figure 12:**
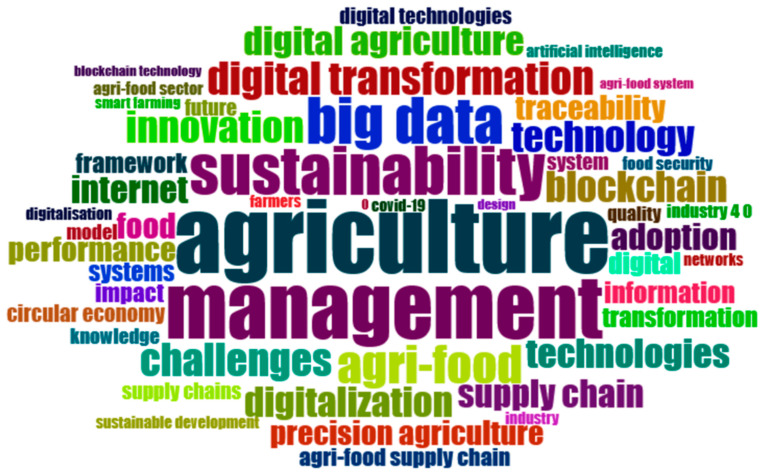
Keyword analysis. Source: Own processing in Bibliometrix.

The *keyword analysis* (Figure 12) underscores the centrality of *agriculture* and *management*—terms with the highest frequencies (90 and 77 occurrences respectively)—which anchor the field’s lexicon in practical applications and organizational governance.

Closely associated terms such as *sustainability*, *big data*, and *agri-food* form the first-order conceptual cluster, reflecting the convergence of data-driven decision-making and environmental objectives. The co-occurrence of *digital transformation*, *blockchain*, and *innovation* indicates an ongoing shift toward technological embeddedness within broader socio-technical systems, where digital infrastructures become important mediators of transparency, traceability, and resilience in food production chains [67,68].

**Figure 13:**
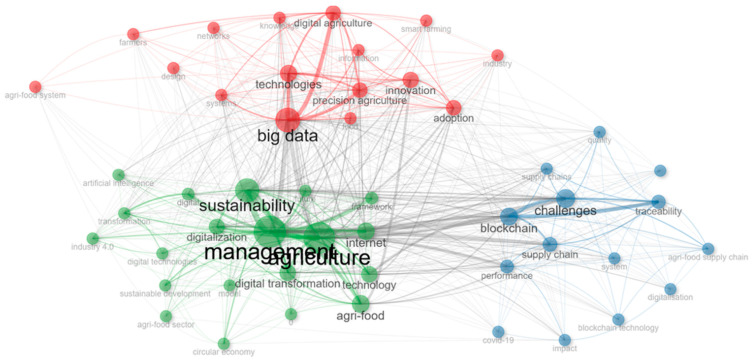
Keyword co-occurrence network. Source: Own processing in Bibliometrix.

Figure 13 presents a *keyword co-occurrence network* that maps the conceptual architecture of scholarly literature at the intersection of digital transformation and sustainability in agri-food systems. The nodes represent recurring keywords, while the links indicate the frequency and strength of co-occurrence relationships. Node size corresponds to the frequency of the term, and node color reflects modularity classes—indicating distinct thematic clusters identified through community detection algorithms.

Three dominant clusters emerged, each representing a core research trajectory:

*The Green Cluster* centered around “sustainability”, “management”, and “digitalization”. This cluster represents the conceptual nucleus of the field, where the discourse is oriented toward integrating technological innovation with sustainable management practices. Terms like digital transformation, sustainable development, agri-food, and circular economy emphasize the systemic nature of change being examined. The high degree of interconnectivity in this cluster suggests a mature, multidisciplinary dialogue linking environmental concerns with digital governance, policy frameworks, and institutional strategies.

*The Red Cluster* concentrated around “big data”, “precision agriculture”, and “digital agriculture”. Located in the upper portion of the network, this group reflects the technological core of the literature. It includes terms such as innovation, adoption, networks, and smart farming, highlighting both technological solutions and their implementation dynamics. The proximity and interlinkage between technologies, information, and knowledge illustrate the theoretical orientation of this subdomain, which focuses on optimizing production systems and enhancing efficiency through data-driven tools.

*The Blue Cluster* is dominated by “challenges”, “blockchain”, and “traceability”. This cluster addresses transparency, logistics, and resilience. It includes keywords like supply chain, performance, quality, impact, and COVID-19, suggesting that this research stream is particularly engaged with risk management, food system vulnerability, and the validation of trust mechanisms through digital technologies. The presence of blockchain as a highly connected node indicates its centrality in the debate on traceability and accountability within complex agri-food supply chains.

Bridging terms such as blockchain, Internet, and digitalization connect the three clusters, functioning as semantic intermediaries that facilitate cross-domain integration. These keywords enable convergence between the governance and ethical dimensions of sustainability (green cluster), the operational and technical imperatives of precision agriculture (red cluster), and the supply chain and risk mitigation concerns (blue cluster).

Overall, the network revealed a thematically mature and structurally cohesive field, wherein digital technologies are not merely standalone tools but are embedded in broader discussions about governance, innovation, and systemic transformation. The overlapping connections across clusters further suggest a strong interdisciplinary orientation and a growing consensus around the role of digitalization as both a technical and institutional driver of sustainable agri-food transitions.

**Figure 14:**
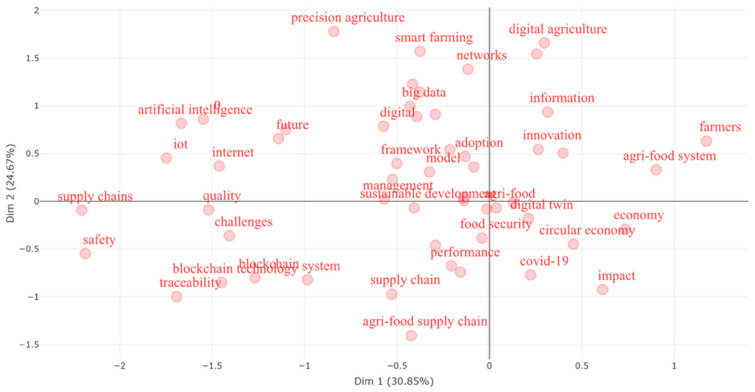
Co-word factorial analysis of articles by cluster. Source: Own processing in Bibliometrix.

Figure 14 illustrates a *co-word factorial analysis of articles by cluster*, a multivariate technique frequently used in bibliometric studies to reduce dimensionality and reveal latent structures within a corpus of scientific literature. The plot positions terms in a two-dimensional space—Dim 1 (30.85%) and Dim 2 (24.67%)—based on their frequency and co-occurrence patterns, thereby uncovering conceptual proximities and thematic relationships in the field of digital agri-food research.

In the *upper-right quadrant*, keywords such as digital agriculture, information, innovation, and agri-food system are clustered together, suggesting a cohesive and active thematic area centered on the technology-driven restructuring of food systems. The proximity of terms like farmers and economy in this region reflects a research orientation toward practical implementation, economic impact, and socio-technical integration of digital tools in agriculture.

Conversely, the *upper-left quadrant* hosts terms like precision agriculture, smart farming, big data, artificial intelligence, and Internet of Things (IoT). These keywords represent a technological subdomain characterized by high computational intensity and technical specificity. Their moderate dispersion suggests a diversity of applications, and their shared position indicates that precision-oriented tools and data-intensive methods form a coherent conceptual cluster within the literature.

The *lower-right quadrant* includes digital twin, circular economy, food security, and COVID-19, which point toward emerging or context-driven topics that bridge sustainability concerns and recent global disruptions. The placement of impact and performance nearby reflects the growing emphasis on evaluative frameworks that assess digitalization outcomes in food systems, especially in light of pandemic-induced vulnerabilities.

In the *lower-left quadrant*, terms such as blockchain, traceability, supply chains, and safety suggest a focus on transparency, logistics, and risk mitigation. The proximity of challenges and quality indicates a significant dimension within the research, engaging with the limitations and governance issues tied to these technological innovations. The relative isolation of these keywords may also reflect a specialized subdomain with strong internal cohesion but weaker integration with broader conceptual themes.

Central to the map are integrative concepts like sustainable development, agri-food, management, model, and adoption, which function as semantic bridges connecting multiple clusters. Their central position underlines their conceptual significance and the frequency with which they co-occur across otherwise distinct thematic areas.

Therefore, this correspondence analysis revealed a field marked by both technological differentiation and conceptual convergence. While specialized clusters (e.g., AI and IoT vs. blockchain and supply chains) indicate methodological diversity, the central positioning of terms related to sustainability, innovation, and management suggests an increasing effort to synthesize technical capabilities with governance and development frameworks. The result is a research landscape that reflects both fragmentation in tools and coherence in transformative purpose.

In synthesis, the intellectual architecture of the field is characterized by *polycentric evolution*—a convergence of technological, managerial, and ethical subdomains. The maturation of digital innovation in agri-food systems thus represents not merely the digitalization of agriculture but the emergence of a new knowledge framework where sustainability, inclusivity, and governance become integral dimensions of technological progress. This conceptual reconfiguration underscores the field’s potential to inform broader sustainability transitions while revealing the persistent need for integrative frameworks that reconcile technological dynamism with social justice and ecological integrity.

### 4.5. Authorial and Citation Dynamics

The analysis of authorial and citation patterns offers relevant insight into the distribution of intellectual influence and productivity within the research landscape on digital innovation in sustainable agri-food systems. The field exhibited a highly networked but asymmetrical authorship structure, where a relatively small number of scholars exerted a disproportionate influence on the evolution of knowledge, while a larger peripheral cohort contributed sporadically to the cumulative discourse. This power-law dynamic conforms closely to Lotka’s Law of scientific productivity, confirming the classical bibliometric observation that a minority of authors generate the majority of impactful contributions.

**Figure 15:**
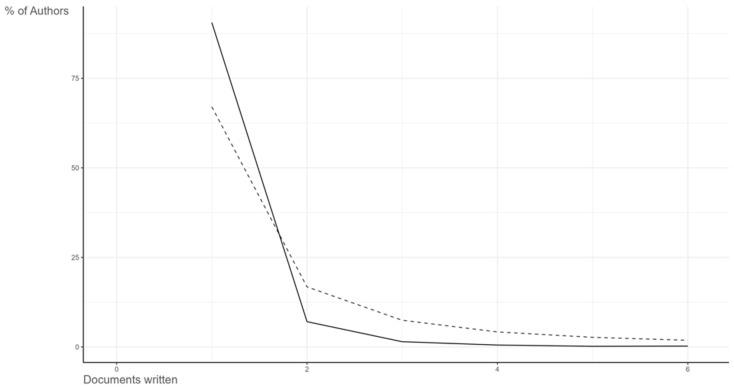
Lotka’s Law distribution. Source: Own processing in Bibliometrix.

The Lotka’s Law distribution (Figure 15) derived from the dataset revealed that approximately 10% of authors were responsible for over 40% of all publications, while the majority were single-contribution researchers. This configuration suggests the presence of an intellectual elite whose sustained output anchors the field’s theoretical and empirical development. Such a concentration of productivity often emerges in young yet rapidly consolidating research areas, where early entrants secure epistemic authority through consistent engagement and network centrality. The dominance of recurring authors not only stabilizes thematic direction but also facilitates methodological standardization, allowing the field to progress from exploratory diversity toward conceptual coherence.

**Figure 16:**
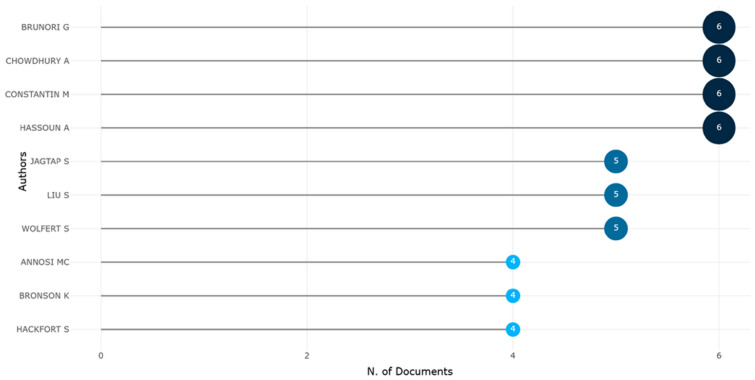
Most locally cited authors. Source: Own processing in Bibliometrix.

Among the *most locally cited authors* (Figure 16), four authors—Brunori G., Chowdhury A., Constantin M., and Hassoun A.—were shown to lead the field with six publications each, indicating sustained engagement and likely centrality in shaping the conceptual foundations and empirical advancements of this interdisciplinary domain.

Just below this leading cohort, authors such as Jagtap S., Liu S., and Wolfert S. emerged with five documents each, reflecting a high level of productivity and probable influence within specialized subfields such as supply chain innovation, digital traceability, or governance frameworks. The appearance of Wolfert S. [69,70] is particularly noteworthy given his established contributions to smart farming and precision agriculture, which have helped bridge technological innovation with sustainability concerns.

Meanwhile, the presence of Annosi M.C., Bronson K., and Hackfort S., with four contributions each, suggests a secondary tier of scholars who, while slightly less prolific, may occupy pivotal roles in niche or emerging thematic clusters such as responsible innovation, socio-political implications of agri-tech, or digital divides.

Overall, the chart underscores the concentration of authorship among a select group of researchers, revealing both a core intellectual leadership and the gradual broadening of scholarly participation. This dynamic not only shapes the research agenda but also contributes to theoretical maturation within the rapidly evolving literature on agri-food digitalization.

These authors’ local citation dominance indicates not only intellectual continuity but also cross-referential coherence, as their works form the conceptual backbone of multiple subsequent studies. The recurrent referencing of these scholars within the dataset suggests the existence of a stable knowledge community, bound by shared theoretical commitments to responsible digitalization and sustainable governance.

**Figure 17:**
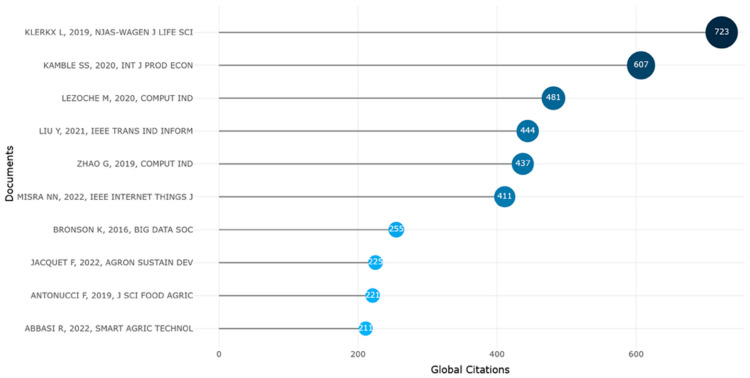
Most globally cited documents. Source: Own processing.

The *most globally cited documents* (Figure 17) analysis further substantiates this concentration of intellectual capital. The document authored by Klerkx L. (2019) stood out prominently, with 723 citations, underscoring its role as a cornerstone in the conceptualization of digitalization processes and governance challenges in agriculture. Its high citation volume indicates widespread recognition and integration into diverse theoretical and empirical investigations.

Following closely, Kamble S.S. (2020) and Lezoche M. (2020) have accumulated 607 and 481 citations respectively, suggesting their substantial influence, particularly in topics related to Industry 4.0 applications, smart agriculture, and the digital supply chain. These works likely serve as methodological or conceptual bridges between operational research and sustainability frameworks.

Contributions by Liu Y. (2021), Zhao G. (2019), and Misra N.N. (2022) also maintained strong citation profiles, reflecting the rising academic interest in the Internet of Things (IoT), artificial intelligence, and digital infrastructure in the agri-food sector. Their inclusion emphasizes the growing cross-disciplinary appeal of digital agriculture, with relevance extending to computer science and industrial informatics.

Mid-tier documents such as those by Bronson K. (2016), Jacquet F. (2022), and Antonucci F. (2019) mark the field’s diversification, addressing pivotal dimensions such as digital ethics, agroecological innovation, and technological adoption. Abbasi R. (2022), while more recent, already shows a notable citation footprint, hinting at the paper’s potential to become a future reference point.

Collectively, this citation distribution reveals a dynamic and maturing research landscape where early foundational texts coexist with emergent contributions, signaling both theoretical consolidation and ongoing innovation in digital agri-food scholarship.

Interestingly, the citation dynamics exhibited a temporal clustering effect, where papers published after 2020 accumulated citations at an accelerated rate compared to earlier works. This pattern suggests a knowledge acceleration phenomenon, wherein new paradigms—especially those addressing circular economy, AI ethics, and digital traceability—rapidly gain traction due to their alignment with global sustainability and digital governance agendas. The citation half-life of these works is relatively short, indicative of a highly reactive and innovation-driven domain where intellectual influence evolves swiftly.

While the dominance of a few high-impact authors and papers reinforces theoretical consolidation, it also exposes potential conceptual vulnerabilities. The reliance on a limited number of conceptual frameworks risks intellectual path dependency, where new research reiterates rather than challenges established paradigms. This phenomenon mirrors trends observed in other emerging interdisciplinary domains, such as smart city studies and digital sustainability, where early frameworks become hegemonic reference points [68,71]. To counteract such homogenization, the field requires diversification through the inclusion of voices from underrepresented regions and methodological traditions—particularly from the Global South and participatory innovation research.

**Figure 18:**
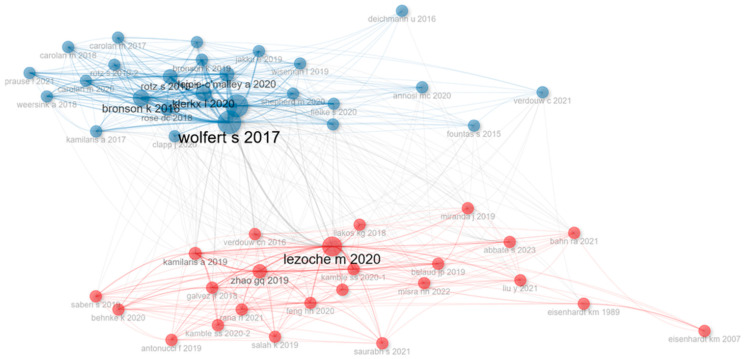
Co-citation network. Source: Own processing in Bibliometrix.

Figure 18 illustrates a *co-citation network* that captures the intellectual structure of agri-food digitalization research by mapping the frequency and strength of simultaneous citations among authors. The visualization revealed two distinct clusters, suggesting the existence of divergent but complementary knowledge domains within the field.

The *blue cluster*, centered around Wolfert S. (2017), represents a foundational school of thought that emphasizes data-driven agriculture, digital infrastructure, and system-level governance. The prominence of authors like Bronson, Klerkx, and Rotz—known for integrating socio-technical frameworks into digital agriculture—indicates a strong alignment with themes such as innovation systems, stakeholder participation, and digital governance. The dense interconnections reflect a cohesive and mature knowledge base, likely grounded in interdisciplinary approaches that merge agricultural science, rural sociology, and information systems.

In contrast, *the red cluster*, led by Lezoche M. (2020), gravitates toward technological implementation, supply chain optimization, and blockchain-based traceability. This domain is more oriented toward operations research, information management, and engineering applications. The co-citation links among works by Zhao, Kamble, Verdouw, and Saurabh suggest a preoccupation with the efficiency, transparency, and resilience of agri-food logistics under digital transformation. The slightly looser interconnections in this cluster may indicate an emerging or more heterogeneous research trajectory, possibly driven by rapidly evolving technologies and case-specific applications.

The limited overlap between clusters suggests that while both domains contribute to understanding digitalization in agri-food systems, they do so from epistemologically distinct perspectives—one focusing on systemic and governance issues, and the other on technical and operational concerns. This structural segmentation highlights the ongoing need for cross-fertilization between socio-institutional and technological discourses to build a more integrative research agenda capable of addressing both strategic and applied dimensions of digital transformation in agriculture.

**Figure 19:**
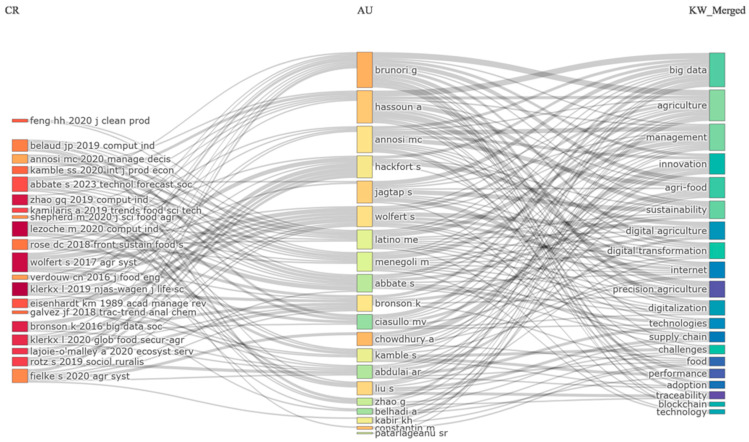
Structural relationships between three components of a bibliometric landscape: the cited references, the contributing authors, and the merged keywords. CR is cited references, AU is the contributing authors, and KW_Merged is the merged keywords that define the thematic orientation of the research domain. Source: Own processing in Bibliometrix.

Furthermore, Figure 19 illustrates the structural relationships between three components of a bibliometric landscape: the cited references, the contributing authors, and the merged keywords that define the thematic orientation of the research domain. The left side groups the core references that form the conceptual and methodological foundation of the field. These works belong primarily to research streams concerned with digital technologies, industrial management, food systems, and supply chain innovation. Their position and the volume of outgoing links indicate that they operate as central sources of theoretical and empirical material used extensively in subsequent studies.

The middle section displays the authors who draw on these foundational works. The density of connections reveals that many authors rely simultaneously on multiple reference nodes, which indicates a shared reliance on established literature. Authors such as Brunori, Hassoun, Annosi, and Wolfert are positioned where the strands converge, showing that their research is strongly embedded in the dominant scholarly conversations about digital transformation and agri-food systems. The spread of authors across the figure also confirms that the field attracts contributions from diverse disciplinary backgrounds, ranging from management and computer science to agricultural studies.

The right side aggregates the keywords associated with the authors’ publications. The distribution of links shows that research activity concentrates on themes such as digital agriculture, precision agriculture, blockchain, traceability, big data, innovation, and sustainability. The consistent flow of connections from authors to these keywords indicates that the field is structured around the integration of digital tools into agri-food supply chains. Keywords like big data, digitalization, traceability, and blockchain are tied to multiple authors, which confirms their central role in shaping current research directions. Other keywords, such as food, performance, or challenges, highlight the applied dimension of these studies, indicating that technological adoption is examined primarily in relation to operational improvements and sustainability pressures.

By linking references, authors, and keywords in a single diagram, the figure shows how foundational studies on digital technologies and food-system management feed directly into contemporary research on data-driven agriculture and supply chain innovation. The convergence of multiple authors toward a shared set of keywords indicates the consolidation of a coherent research domain, where digital transformation in agri-food systems is the principal axis connecting prior knowledge, current scholarly activity, and thematic priorities.

Overall, the authorial and citation structures delineate a research field that has moved beyond fragmentation toward structured interdependence. A network of core authors now provides conceptual stability, while citation flows reveal a cumulative, self-reinforcing knowledge ecology characterized by rapid theoretical adaptation and expanding policy relevance. The interplay between intellectual concentration and thematic diversification constitutes both the field’s strength and its frontier: the capacity to sustain coherence without succumbing to theoretical closure.

### 4.6. Thematic Evolution and Emerging Research Frontiers

The thematic evolution of research on digital innovation for sustainable agri-food systems reveals a profound knowledge transformation, moving from a focus on technological capability toward an emphasis on governance, ethics, and systemic resilience. The analysis of trend topics and keyword growth trajectories illustrates that the field has undergone several waves of conceptual expansion, each driven by different socio-technical imperatives and global sustainability agendas.

**Figure 20:**
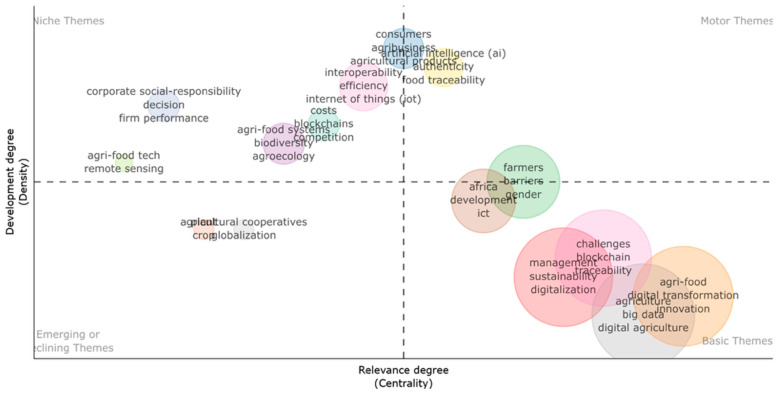
Strategic thematic map. Source: Own processing in Bibliometrix.

Figure 20 represents a *strategic thematic map* commonly used in bibliometric analysis to visualize the positioning of research themes based on two key dimensions: centrality (indicating relevance within the overall scientific field) and density (indicating the level of internal development of each theme). The quadrants reflect distinct types of thematic significance and maturity, thereby offering insight into the intellectual and structural configuration of the literature on digitalization in agri-food systems.

In the upper-right quadrant, labelled as *Motor Themes*, we observed clusters such as food traceability, agricultural productivity, authenticity, and artificial intelligence (AI). These topics are characterized by both high centrality and high density, signifying that they are not only well-developed within their thematic area but are also highly relevant to the broader discourse. Their position suggests that they function as conceptual and methodological drivers of the field, playing a central role in the digital transformation of agri-food systems.

Moving to the lower-right quadrant, which represents *Basic and Transversal Themes*, we find prominent concepts such as digital agriculture, agriculture, big data, blockchain, and sustainability. Despite having relatively lower internal development compared to motor themes, their high centrality implies that these are foundational constructs underpinning much of the current research. This quadrant typically includes themes that are broadly cited and conceptually integrated but may still require deeper theoretical refinement.

In the upper-left quadrant, labelled as *Niche Themes*, the figure includes clusters like corporate social responsibility, firm performance, and remote sensing. These themes display strong internal cohesion but limited external connectivity. Their relative isolation indicates specialized areas of inquiry that are methodologically mature but peripheral to the dominant structure of the field. They may serve as important areas of application or methodological refinement, albeit with limited cross-thematic influence.

The lower-left quadrant, which denotes *Emerging or Declining Themes*, contains terms such as agricultural cooperatives, crop globalization, and agri-food tech. These themes exhibit both low centrality and density, suggesting that they either represent emerging areas of inquiry yet to be fully integrated into the core research agenda or declining topics losing traction within the academic community. The positioning invites further investigation into whether these clusters are undergoing conceptual renewal or are becoming obsolete.

Additionally, the presence of context-specific themes such as Africa, gender, ICT, and development—moderately placed between basic and motor themes—highlights an important regional and social dimension within the literature. These clusters, while not yet fully central or dense, point to an expanding focus on inclusivity, equity, and technological capacity building in underrepresented contexts.

Overall, the thematic map illustrates the multi-layered structure of the agri-food digitalization research field. It underscores the coexistence of mature, central paradigms (e.g., traceability, big data, digital agriculture), rapidly advancing thematic cores (e.g., AI, authenticity), and emerging socially embedded challenges (e.g., gender, ICT in Africa). This distribution reflects a field in transition—where technological sophistication is increasingly accompanied by normative concerns related to governance, participation, and sustainability.

Therefore, the current landscape reveals a fragmented conceptual space, where high-impact sustainability discourse is still relatively disconnected from the technological backbone of digital agriculture. Bridging these gaps may involve integrating socio-institutional themes (e.g., equity, governance, data sovereignty) into the more technical literature on automation and precision systems. This figure calls attention to the need for cross-domain synthesis, where impactful themes like blockchain and sustainability become more central to the design and evaluation of agri-digital infrastructures. Therefore, this visualization underscores the asymmetry between normative influence and systemic integration, offering a diagnostic lens for researchers and policymakers seeking to align technological innovation with sustainable agri-food transformation.

During the *initial stage (2004–2014),* the discourse was dominated by themes such as *precision agriculture*, *ICT*, and *automation*. These early studies were largely technocentric, aiming to enhance productivity and resource efficiency through the deployment of sensors, geographic information systems, and mechanized data collection. This phase corresponded with the broader narrative of agricultural modernization, in which sustainability was treated primarily as a by-product of efficiency rather than as a guiding principle of technological design.

The *transitional phase (2015–2020)* introduced a shift toward systemic and integrative perspectives, with emerging topics including *digital transformation*, *innovation ecosystems*, and *big data analytics*. This evolution was catalyzed by the institutionalization of sustainability targets through frameworks such as the Paris Agreement (2015) and the UN Sustainable Development Goals (2016). Consequently, the literature began to merge digitalization with sustainability theory, positioning technologies as both tools and mediators of systemic change. This period also marked the introduction of the circular economy paradigm, which reframed resource optimization within regenerative and restorative economic logics.

The most recent *maturity phase (2021–2026)* indicates an epistemological deepening of the field. Trend topic mapping identified a surge in concepts such as *blockchain*, *traceability*, *AI ethics*, *digital twins*, and *data governance*. The recurrence of these terms signals a shift from *digitalization for efficiency* to *digitalization for accountability and transparency*. Digital technologies are no longer portrayed as neutral instruments but as actors within complex socio-technical assemblages whose governance determines their contribution to sustainability [4,12]. This transformation reflects the growing awareness of data justice, algorithmic bias, and equity in digital innovation, integrating sustainability with ethics and inclusivity.

The temporal density of emerging keywords indicates that from 2022 onward, the discourse increasingly centers on *resilience*, *circular economy*, and *smart governance*. This evolution parallels the transition from supply chain optimization to value chain reconfiguration, where the focus shifts from isolated efficiency metrics to systemic transformation. Moreover, *resilience* has become a dominant thematic axis, particularly after the COVID-19 pandemic, as scholars and policymakers alike recognized the fragility of global agri-food networks and the need for adaptive, digitally supported responses.

The emergence of blockchain-related research represents one of the most distinctive frontiers. Initially conceptualized as a technological tool for ensuring product authenticity and provenance, blockchain has since evolved into a socio-technical governance mechanism for trust-building and transparency. Studies by Kamble et al. [67] and Annosi et al. [72] have indicated that distributed ledger technologies can restructure inter-organizational relationships, but only when coupled with ethical and inclusive governance models. Thus, blockchain research within this field is increasingly aligned with normative theories of responsible innovation, emphasizing trust, inclusivity, and participatory design.

Another notable trajectory is the integration of artificial intelligence and predictive analytics into sustainability frameworks. The proliferation of research on *digital twins* and *AI-enabled decision support systems* reveals a growing ambition to simulate ecological complexity and predict system-level responses. However, these developments have also reignited important debates around technological sovereignty and ethical design, as algorithmic decision-making risks consolidating power among technologically advanced actors, particularly within data-rich agribusiness contexts [73].

At the intersection of governance and ethics, the field has begun to embrace post-digital sustainability frameworks that conceptualize technology as embedded within socio-political and ecological systems. This marks a radical epistemological departure from earlier paradigms: digital technologies are now examined through the lens of sustainability transitions, knowledge co-production, and reflexive governance. These frameworks resonate with broader theoretical currents in sustainability science, particularly the *transformative innovation policy* perspective [74], which emphasizes collective learning, distributed responsibility, and long-term societal benefit.

Despite this conceptual sophistication, the field remains marked by *regional and thematic asymmetries.* The majority of studies originate from high-income regions, where digital infrastructure and institutional capacity are well-established, leaving knowledge gaps in areas such as smallholder digital adoption, data governance in low-resource settings, and gendered dimensions of digital transformation. These omissions delineate critical frontiers for future inquiry.

From a comparative perspective, the thematic evolution observed here aligns with trends identified in adjacent domains such as smart cities [71] and industrial sustainability [68], where research trajectories similarly move from digital integration toward governance reflexivity. However, the agri-food context remains distinct due to its material embeddedness in ecological cycles and food security imperatives. Digital innovation in this sector thus operates under dual constraints: it must deliver both technological functionality and ecological legitimacy.

In synthesis, the thematic and trend analyses reveal a field in active transition toward a reflexive and governance-oriented paradigm. Future research is expected to expand along three converging axes: (i) ethical and regulatory frameworks for responsible digitalization, (ii) integration of AI and blockchain in transparent and circular value chains, and (iii) inclusive innovation models addressing regional disparities in digital capacity. Together, these frontiers define the next knowledge horizon of digital sustainability in agri-food systems—a domain where technological advancement, social equity, and ecological stewardship are not competing priorities but interdependent conditions for transformation.

## 5. Discussion

### 5.1. Comparative Insights from Prior Bibliometric and Empirical Studies

The findings of this study outline the extent to which digitalization has reshaped the intellectual contours of research on sustainable agri-food systems. The thematic evolution detected through co-word and temporal analyses indicates a clear transition from early preoccupations with technological efficiency toward a more governance-oriented and ethically grounded understanding of digital transformation. This shift is reflected in the consolidation of four major thematic axes—digital governance, blockchain-based traceability, circular economy integration, and ESG performance metrics—each of which contributes to a reframing of sustainability as a digital domain. The patterns uncovered thus reveal that digital technologies no longer operate as isolated tools but have become embedded in broader conceptual frameworks that redefine transparency, value creation, and accountability within agri-food systems. This conceptual reorientation aligns closely with the first research question by showing how digital infrastructures have triggered a substantive transformation in the thematic landscape.

The structure of scholarly collaborations reinforces this thematic realignment. Bibliographic coupling maps indicate that distinct clusters of researchers have coalesced around the newly emergent thematic axes, forming coordinated knowledge communities that advance digital governance, traceability technologies, circularity principles, or ESG frameworks. These collaborative patterns reveal a field that is no longer fragmented but increasingly consolidated through interdisciplinary linkages. The convergence of researchers from management, engineering, computer science, and sustainability studies indicates that digitalization has functioned as a bridging mechanism that strengthens the integrative character of agri-food scholarship. In this respect, the study contributes to understanding how collaboration networks support the consolidation of interdisciplinary agendas and offer a clearer view of the intellectual communities shaping the digital–sustainability nexus.

Institutional dynamics further clarify the drivers behind this consolidation. The analysis highlights the pivotal role played by European research centers—particularly those in Italy, France, and the Netherlands—in setting the agenda for digital sustainability in agri-food systems. The dominance of these institutions is strongly aligned with the presence of ambitious policy frameworks such as the European Green Deal and the Farm-to-Fork Strategy, suggesting that institutional environments with strong policy support tend to cultivate high-impact research in digital transformation. These results indicate that research centers differ not only in productivity but also in thematic orientation, with European institutions acting as incubators for governance-oriented and digitalization models. Such institutional differentiation contributes to the global patterning of research agendas and supports a more nuanced understanding of how digitalization is framed within different academic and policy contexts.

The geographical distribution of publications and cooperation ties provides additional insight into how regional innovation systems shape the international structure of the field. With Europe emerging as the leading knowledge hub, the results underscore how regional capacities for digital innovation and sustainability governance influence patterns of global collaboration. The concentration of output in European contexts suggests that regulatory ambition and investment in digital infrastructures translate into strong academic leadership. In contrast, regions with less developed digital ecosystems appear less represented, reflecting structural asymmetries in access to digital tools and scientific resources. These findings thus shed light on how geopolitical and policy landscapes mediate the global circulation of knowledge in agri-food digitalization.

The implications of these results extend beyond descriptive mapping. By conceptualizing digitalization as both technological infrastructure and socio-institutional mechanism, the study points toward the need for future scholarship to develop integrated analytical frameworks capable of linking digital infrastructures with sustainability transitions and broader institutional change. Such frameworks should account for the interdependence between data governance, interoperability standards, participatory models of decision-making, and the socio-political dimensions of digital adoption. The identification of persistent digital divides and imbalance in knowledge legitimacy suggests that future research should prioritize inclusivity and reflexivity, addressing the uneven distribution of knowledge and capacities that currently shape the field.

Thus, the results position digitalization not as a mere technical upgrade but as a profound theoretical reconfiguration of how sustainability is conceptualized, operationalized, and governed within agri-food systems. The integration of thematic evolution, collaborative structures, institutional dynamics, and geographic patterns provides a comprehensive understanding of this transformation and delineates clear avenues for advancing both theory and practice in the digital era of sustainable agriculture.

A relevant comparison with related domains strengthens these conclusions. In the smart city literature, Komninos [71] observed a similar dialectic between technological determinism and participatory governance. However, the agri-food sector exhibits a unique bio-physical embeddedness, where digital innovation directly interacts with ecological processes. This creates a distinctive tension: technologies designed to increase efficiency may simultaneously intensify resource use or centralize control. The recent literature on ethical AI and data sovereignty [73,75,76,77,78,79] explicitly addresses this paradox, emphasizing that the sustainability of digital innovation depends on how power, data, and value are distributed along the agri-food chain.

Nevertheless, despite its increasing sophistication, the field still faces significant research gaps. Most notably, empirical studies remain concentrated in Europe and high-income economies, while the digital transformation of smallholder agriculture in the Global South is comparatively underexplored. This imbalance risks reinforcing global knowledge asymmetries and technological dependencies [80]. Similarly, the gendered and social dimensions of digital adoption—highlighted in studies such as Kitta et al. [81]—remain peripheral to the mainstream discourse.

From a theoretical perspective, this study confirms that the conceptual trajectory of the field aligns with reflexive modernization theory [82], wherein technological advancement generates not only solutions but also new risks that require continuous institutional adaptation. Digital innovation in agri-food systems thus becomes both a driver of modernization and a subject of reflexivity—an object of governance as much as an instrument of it.

Thus, the discussion highlights that the literature on sustainable agri-food systems through digital innovation is entering a post-optimistic phase, characterized by critical engagement with power, ethics, and governance. Unlike earlier narratives of digital utopianism, the current research agenda emphasizes responsibility, resilience, and inclusivity as prerequisites for sustainable digital transitions. This intellectual reorientation positions the field as a leading frontier within sustainability science, capable of informing both academic inquiry and policy design toward a more equitable and transparent digital future.

Moreover, the patterns emerging from the present bibliometric analysis broadly align with the trajectory described in recent agrifood and sustainability research, yet they also extend the field’s understanding in several significant respects. The results confirm that digital transformation in the agri-food domain has shifted from isolated technological experimentation toward complex governance-oriented configurations, echoing the conceptual framework proposed by Wolfert et al. [68,69]. Their work conceptualized digital innovation systems as multi-actor arrangements in which infrastructure, data governance, and policy co-evolve; the co-authorship and institutional networks identified in this study empirically validate that vision, revealing Europe—particularly Italy, Spain, and the Netherlands—as the principal locus of such integrative activity. Nevertheless, while Wolfert and colleagues remained largely theoretical, the present results quantify the diffusion of digital sustainability discourse through global collaborations, thereby offering concrete evidence of the systemic maturity of digital agrifood research.

A similar resonance is observed with the empirical conclusions of Zarbà et al. [29], who documented how blockchain has transformed from a traceability instrument into a mechanism for value co-creation and circular economy verification. Our keyword co-occurrence and thematic evolution data confirm this conceptual migration but also highlight an accelerating interdisciplinarity that exceeds Zarbà’s operational lens. Whereas their case studies emphasize the technical affordances of blockchain, the current results show how its symbolic association with “transparency”, “governance”, and “ESG” has repositioned it at the heart of sustainability-oriented digitalization debates. The conceptual broadening captured bibliometrically thus represents an epistemic shift: blockchain is now less a technological artifact than a coordinating institution within sustainable food networks.

The comparative reading of policy-oriented studies further supports this interpretation. Reinhardt [73] evaluated the EU’s Farm-to-Fork strategy through the lens of innovation systems, contending that digitalization policies yield impact only when they foster vertical and horizontal coordination among diverse actors. The country-level scientific production and international collaboration patterns observed in our data affirm this assertion. However, the bibliometric evidence adds granularity by revealing a temporal coupling between EU funding cycles and publication surges, demonstrating empirically what Reinhardt theorized—the dependence of digital transition momentum on policy synchronization.

At the organizational level, Annosi et al. [48,72] converged on the argument that managerial cognition and institutional capability remain decisive for successful digital transformation. The present analysis corroborates their observations: terms such as “capability”, “innovation”, and “organizational change” cluster prominently in highly cited works, signifying their conceptual centrality. Yet, in contrast to these firm-level studies, our findings expose the global asymmetry of capability diffusion. While European and East Asian networks generate dense innovation linkages, the Global South continues to appear peripherally in co-authorship and thematic maps—a structural imbalance that micro-level analyses cannot capture.

These disparities are also reflected in the emergent resilience and ESG literatures. Camel et al. [42] demonstrated through quantitative modeling that dynamic digital capabilities enhance supply-chain resilience, particularly after exogenous shocks such as the COVID-19 pandemic. Correspondingly, “resilience” emerged in our thematic trend analysis as a post-2021 keyword of growing influence. Our bibliometric evidence not only confirms these relationships but also adds temporal precision: ESG terminology proliferated later—around 2023—but grew exponentially, particularly in economies with established digital infrastructures and standardized disclosure frameworks. Such temporal patterning suggests that ESG digitalization constitutes the latest conceptual frontier in the field’s evolution.

Equally notable is the convergence between our thematic clusters and Sica et al. [25]’s integration of life cycle assessment with digital traceability. Both bodies of evidence highlight the fusion of environmental accountability with digital data infrastructures. However, our global co-word network expands this insight by showing how the LCA–traceability nexus transcends environmental science, permeating management and information systems research. This cross-disciplinary migration indicates a more holistic conceptualization of sustainability—one that unites technical assessment, organizational learning, and ethical accountability within a single digital ecosystem.

Nevertheless, not all trajectories point toward convergence. The growing attention to critical perspectives, exemplified by Guthman and Fairbairn [45], introduces an important countercurrent. These authors interrogate the socio-political consequences of agri-tech, particularly the reconfiguration of labor regimes, data ownership, and rural agency. Our co-word clusters, where “labor”, “equity”, and “inclusion” begin to intersect with “digitalization”, demonstrate that this critical discourse is now permeating mainstream research rather than remaining marginal. Yet, unlike the qualitative depth of Guthman’s ethnographic work, our bibliometric mapping offers a macroscopic confirmation that such concerns are gaining institutional legitimacy and citation influence, signaling a maturing debate between techno-optimist and critical-political strands.

The comparative scope widens further when governance-oriented digitalization is considered. van Wassenaer et al. [30] and Wolfert et al. [70] emphasize that sustainability outcomes depend less on the technologies themselves than on the architectures of governance that mediate their use. This is consistent with our finding that “governance” functions as a cross-cutting hub within the keyword network and appears disproportionately in high-impact papers. However, our study adds institutional geography to this argument: governance-centric research originates predominantly in European and OECD contexts, whereas technical and operational studies cluster in emerging economies, a divergence with implications for global policy harmonization.

Finally, when viewed through the lens of information integrity and societal trust, our results resonate with the observations of Chowdhury et al. [83], who caution that digital expansion can exacerbate misinformation and inequity. While their study is micro-contextual, focused on Sri Lanka, our bibliometric evidence reveals a broader epistemic concern—one in which “misinformation”, “trust”, and “transparency” become increasingly co-located in recent publication clusters. This confirms that the debate has shifted from digital capability to digital credibility, reflecting a field in epistemological transition.

Taken together, these comparisons demonstrate that our bibliometric investigation not only validates established theoretical expectations but also enriches them with macro-level empirical substantiation. By quantifying thematic evolution, institutional geography, and collaboration intensity, the study extends the prior literature that was often fragmented across disciplines or confined to single-country analyses. Moreover, by identifying emerging conceptual linkages—such as the convergence of ESG, blockchain, and governance—the findings reveal an epistemic consolidation around the idea of sustainable digital governance. In contrast to prior case-based or normative works, our contribution lies in integrating these diverse streams into a coherent global synthesis, positioning the agrifood digitalization debate at the intersection of technological innovation, institutional coordination, and social accountability.

### 5.2. Regional Asymmetries in Digital Agriculture: Scientific Output Versus Field-Level Dynamics

To support such an analysis, comparative evidence is already available. A global survey from 2022 of 5500 row- and specialty-crop farmers indicates that agriculture technology (agtech) adoption differs sharply across regions: Europe and North America lead, with about 61% of farmers currently using or planning to adopt at least one agtech product within the next two years, while adoption is lowest in Asia (about 9%) [84]. The same source reports meaningful within-region variation; for example, in Europe, agribusiness marketplace adoption is highest in Germany (13%), whereas precision-agriculture hardware usage is highest in the Netherlands (33%). In Asia, surveyed farmers in India reportedly use more agtech products than those in China across the submarkets assessed [84]. These comparisons underline that agricultural digitalization varies substantially across countries and regions, even when countries are grouped under the same broad geography [84].

Such differences also highlight the need to align bibliometric indicators (e.g., publication or patent counts by country) with empirical realities in the field. Evidence syntheses emphasize that adoption effects and uptake patterns depend on technologies, commodity systems, farm characteristics, and local contexts—meaning that “output” measured in publications or patents may not map cleanly onto diffusion at the farm level without additional, ground-truthing evidence [85].

To complement bibliometric trends, empirical investigations are therefore necessary. Case studies can capture real-world impacts and identify adoption constraints and success factors—frequently linked to connectivity and infrastructure, up-front and ongoing costs, skills and training, usability, and perceived return on investment [85,86]. This type of evidence can clarify why some innovations scale and others remain confined to pilots or niche user groups.

In addition, analyzing national policy frameworks for digital agriculture can add explanatory power. India offers a relevant example of policy-driven experimentation: in September 2021, India’s Ministry of Agriculture and Farmers’ Welfare signed multiple memorandums of understanding (MoUs) for pilot projects under its Digital Agriculture Mission (2021–2025), explicitly framing pilots around technologies such as artificial intelligence, blockchain, remote sensing and geographic information system technology (GIS), drones, and robotics [87]. More recently, the Government of India (via a Cabinet Committee decision) approved a “Digital Agriculture Mission” (announced publicly on 4 September 2024, approval dated 2 September 2024) with a dedicated financial outlay and a design centered on digital public infrastructure (including AgriStack and the Krishi Decision Support System—Krishi-DSS is the geospatial platform of Department of Agriculture and Farmers Welfare of Ministry of Agriculture and Farmers Welfare, Government of India), alongside initiatives such as digital crop estimation and soil profile mapping [88,89]. Read together, these policy signals illustrate how national strategies may evolve from pilot partnerships toward scaled infrastructure and governance, which can then be evaluated against adoption and outcomes on the ground [87,89].

Nigeria provides another instructive case: Nigeria’s Digital Agriculture Strategy is commonly described in the literature as a ten-year plan (2020–2030) intended to guide the adoption of digital technologies in agriculture and is discussed as part of broader digital-agriculture policy development in West Africa [90]. A policy repository also records a “Draft Nigeria Digital Agriculture Strategy (2020–2030)” (current version dated January 2022), positioning it as a ten-year roadmap for digital technology adoption in the agricultural sector [90,91]. Likewise, Kenya’s DigiFarm platform has issued tens of thousands of micro-loans to smallholders via mobile apps [92]. Such case studies demonstrate how digital tools are being used by farmers and governments, highlighting successes and obstacles in implementation. By discussing these concrete examples, the analysis acknowledges how technology adoption plays out in practice—from small-scale pilot projects to large national programs—offering insights beyond what publication metrics can tell.

In addition, examining national policy frameworks and evaluations is essential for understanding regional asymmetries. Different countries adopt digital agriculture at different paces often because of policy support or lack thereof. Many governments now have dedicated strategies to promote digital farming, and evaluating these policies reveals their impact. For example, Ethiopia’s Digital Agriculture Roadmap (launched in 2025) sets ambitious targets: reaching 30 million farmers with digital services and raising farm incomes by 8% within five years [92]. Early investment under this plan (over $90 million) underscores a strong political will to drive adoption in a developing country context.

At the EU level, policy direction similarly points toward mainstreaming digital and data-driven approaches. The European Commission frames digitalization as central to agriculture and rural development, including work toward a common European agricultural data space [93]. The Farm-to-Fork strategy explicitly links sustainability and competitiveness objectives to the development of a common European agriculture data space as part of the EU data strategy [94]. In parallel, Common Agricultural Policy (CAP)-focused evaluation work notes that the 2023–2027 CAP gives more attention to digitalization and foresees that Member States elaborate digital strategies to increase the adoption of digital technologies in agriculture and rural areas [95].

Moreover, even within the same continent, significant disparities emerge: South America’s overall agtech uptake is roughly 50%, but Brazil and Argentina considerably outpace countries like Chile in adopting precision farming technologies [96]. These gaps imply that factors like cost, farmer training, service availability, and perceived benefits strongly influence adoption [96]. Therefore, this strengthened regional analysis discusses such cross-country differences in technology use, illustrating how local context and challenges shape the deployment of digital tools on farms.

## 6. Conclusions

The present bibliometric investigation into sustainable agri-food systems through digital innovation offers a comprehensive and data-driven portrait of a rapidly evolving research field that has matured both conceptually and methodologically between 2004 and 2025. By systematically analyzing publications indexed in the Web of Science and deploying Bibliometrix techniques, this study uncovered the knowledge architecture, thematic trajectories, and intellectual power structures underpinning this interdisciplinary domain.

At a conceptual level, the results indicate that digital innovation has become inseparable from sustainability discourse in agri-food systems. What began as a technologically deterministic narrative centered on efficiency and productivity has evolved into a reflexive, governance-oriented paradigm emphasizing accountability, inclusivity, and ethical responsibility. This transformation signifies a paradigmatic realignment: digitalization is no longer merely an enabler of agricultural modernization but a constitutive element of sustainability transitions. The field’s intellectual consolidation around clusters such as digital transformation, blockchain, AI, and circular economy attests to the deep interlinkage between technological innovation and ecological resilience.

From a *theoretical standpoint*, the findings contribute to the advancement of sustainability transition theory by demonstrating that digital technologies operate not only as tools of optimization but also as institutional catalysts reshaping the governance of food systems. The coupling analysis revealed the formation of an epistemic lineage connecting innovation systems theory [13] with the ethics of digital sustainability [12], suggesting the emergence of a hybrid theoretical framework that bridges technological systems with socio-ecological governance. This hybridization supports the argument that the digital transformation of agri-food systems is best understood as a co-evolutionary process, wherein technology, institutions, and sustainability goals mutually reinforce or constrain one another.

Moreover, the study advances bibliometric knowledge by identifying the non-linear maturation dynamics of this research field. The temporal and trend analyses revealed an accelerating rate of conceptual innovation post-2020, coinciding with global crises such as the COVID-19 pandemic, the European Green Deal, and the digitalization imperatives of the Farm-to-Fork Strategy. These exogenous events acted as catalysts for knowledge systems restructuring, intensifying research around resilience, traceability, and AI ethics. The resulting knowledge ecosystem reflects both reactive adaptation—addressing immediate disruptions—and proactive transformation, aimed at systemic redesign.

The theoretical implications of these findings are multifaceted. First, they confirm the growing validity of a socio-technical systems perspective, which understands technological change as embedded within political, economic, and cultural structures. Second, they underscore the relevance of reflexive modernization [80], wherein technological advancement generates new forms of uncertainty and governance demand. Third, they extend transformative innovation policy theory [73] by demonstrating that digital technologies can function as both enablers and regulators of sustainability transitions, depending on institutional design and stakeholder inclusion.

The *practical implications* are equally significant. For policymakers, the findings highlight the necessity of designing integrated digital governance frameworks that balance innovation incentives with ethical and environmental safeguards. Digitalization strategies must move beyond efficiency metrics to embrace data transparency, participatory decision-making, and social inclusion. For practitioners and agribusiness actors, the study emphasizes the importance of responsible digital adoption—ensuring that technologies such as blockchain, IoT, and AI enhance not only productivity but also fairness and trust across value chains. For researchers, the bibliometric mapping provides a navigational tool for identifying thematic clusters, collaboration networks, and underexplored intersections between sustainability, data ethics, and policy integration.

Nevertheless, this study also recognizes its *limitations*, which open avenues for future exploration. First, the analysis is limited to Web of Science data and thus may underrepresent regional or non-English publications that contribute substantially to local innovation ecosystems. Second, the bibliometric approach, while revealing macro-structural patterns, cannot fully capture the interpretive depth and contextual variability of individual case studies. Third, the citation-based metrics privilege academic visibility over practical impact, potentially overlooking innovations implemented outside of scholarly circulation.

Building on these limitations, several *future research directions* emerge. Scholars should deepen investigation into context-specific digital adoption, particularly among smallholder farmers and marginalized regions where technological infrastructures remain uneven. There is also a pressing need for comparative research across governance regimes to understand how policy frameworks mediate digital sustainability outcomes. Furthermore, the intersection of AI ethics, data sovereignty, and food security constitutes a pivotal frontier for both empirical and theoretical development. Finally, methodological innovation is required to integrate bibliometric, qualitative, and participatory research, enabling a more holistic understanding of how digitalization interacts with socio-ecological complexity.

In conclusion, this study provides a rigorous empirical foundation for the ongoing theorization of digital transformation within sustainable agri-food systems. It indicates that the field is undergoing profound theoretical reconfiguration—from a phase of technological euphoria to a period of reflexive equilibrium, where questions of justice, transparency, and governance are as central as those of efficiency and productivity. By bridging bibliometric evidence with conceptual synthesis, this paper advances both the science of sustainability transitions and the policy discourse surrounding responsible digitalization. Ultimately, the study positions digital innovation not as a mere instrument of modernization but as a transformative force whose governance will define the contours of a sustainable, equitable, and intelligent food future.

## Data Availability

All data were obtained from the Web of Science Core Collection Database. Publicly available datasets were analyzed in this study. These data can be found here: https://0c10qjxkk-y-https-www-webofscience-com.z.e-nformation.ro/wos/woscc/basic-search (accessed on 15 October 2025).
